# A Nutrigenetic Update on *CETP* Gene–Diet Interactions on Lipid-Related Outcomes

**DOI:** 10.1007/s11883-022-00987-y

**Published:** 2022-01-31

**Authors:** Ramatu Wuni, Gunter G. C. Kuhnle, Alexandra Azzari Wynn-Jones, Karani Santhanakrishnan Vimaleswaran

**Affiliations:** 1 Hugh Sinclair Unit of Human Nutrition, Department of Food and Nutritional Sciences, University of Reading, Reading, RG6 6DZ UK; 2 The Institute for Food, Nutrition, and Health (IFNH), University of Reading, Reading, UK

**Keywords:** Cholesteryl ester transfer protein, Polymorphisms, Diet, Lipids, Genetic Epidemiology

## Abstract

**Purpose of Review:**

An abnormal lipid profile is considered a main risk factor for cardiovascular diseases and evidence suggests that single nucleotide polymorphisms (SNPs) in the cholesteryl ester transfer protein (*CETP*) gene contribute to variations in lipid levels in response to dietary intake. The objective of this review was to identify and discuss nutrigenetic studies assessing the interactions between *CETP* SNPs and dietary factors on blood lipids.

**Recent Findings:**

Relevant articles were obtained through a literature search of PubMed and Google Scholar through to July 2021. An article was included if it examined an interaction between *CETP* SNPs and dietary factors on blood lipids. From 49 eligible nutrigenetic studies, 27 studies reported significant interactions between 8 *CETP* SNPs and 17 dietary factors on blood lipids in 18 ethnicities. The discrepancies in the study findings could be attributed to genetic heterogeneity, and differences in sample size, study design, lifestyle and measurement of dietary intake. The most extensively studied ethnicities were those of Caucasian populations and majority of the studies reported an interaction with dietary fat intake. The rs708272 (TaqIB) was the most widely studied *CETP* SNP, where ‘B1’ allele was associated with higher *CETP* activity, resulting in lower high-density lipoprotein cholesterol and higher serum triglycerides under the influence of high dietary fat intake.

**Summary:**

Overall, the findings suggest that *CETP* SNPs might alter blood lipid profiles by modifying responses to diet, but further large studies in multiple ethnic groups are warranted to identify individuals at risk of adverse lipid response to diet.

**Supplementary Information:**

The online version contains supplementary material available at 10.1007/s11883-022-00987-y.

## Background

**Fig. 1:**
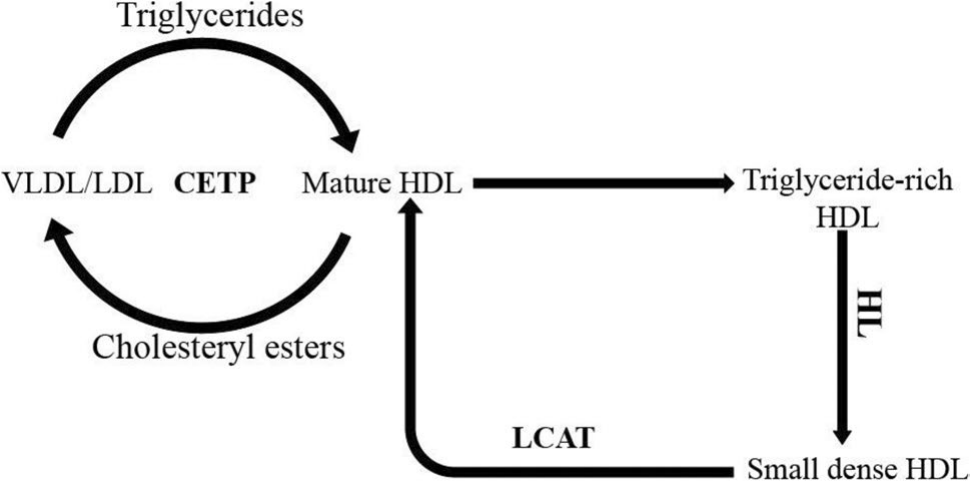
The role of cholesteryl ester transfer protein in lipid metabolism

The global burden of cardiovascular diseases (CVDs) is well recognised and ischaemic heart disease alone accounted for 9 million deaths in 2019, making it the top cause of death in all parts of the world [1]. An abnormal lipid profile (dyslipidaemia), indicated by low concentrations of high-density lipoprotein cholesterol (HDL) and elevated levels of low-density lipoprotein cholesterol (LDL) or triglycerides (TG), is considered a major risk factor for CVDs [2, 3]. The cardioprotective role of HDL is thought to be dependent on the function of HDL rather than the levels of HDL, which is reflected in individuals with scavenger receptor class B member 1 (*SCARB1*) gene mutations who have higher levels of HDL but higher CVD risk [4]. There is evidence to suggest that a combination of genetic susceptibility and environmental factors including diet is responsible for CVDs [5••, 6, 7]. Single nucleotide polymorphisms (SNPs) in lipid-related genes such as the cholesteryl ester transfer protein (*CETP*), lipoprotein lipase (*LPL*) and apolipoprotein E (*ApoE*) genes have been found to contribute to changes in lipid profiles in response to diet [8, 9, 10•]. Of these three genes, *CETP* has been shown to have more associations with blood lipids (Supplemental Table 1). *CETP* regulates the concentration and particle size of HDL cholesterol in the plasma (Fig. 1) and is considered to play an important role in reverse cholesterol transport which is a protective mechanism against atherosclerosis [11]. Increased *CETP* activity has been shown to result in lower HDL levels and is linked to higher risk of CVDs [12].

Several studies have demonstrated *CETP*–diet interactions on blood lipids; however, the findings have been inconsistent [10•, 13–17]. The objective of this review was therefore to identify and discuss studies assessing the interactions between *CETP* SNPs and dietary factors on blood lipids and to identify the factors that can be attributed to these discrepancies.

Cholesteryl ester transfer protein (*CETP*) is a plasma glycoprotein which is secreted by the liver and is responsible for transporting cholesteryl esters and triglycerides between HDL and apolipoprotein B–containing lipoproteins such as very-low-density lipoprotein (VLDL) and low-density lipoprotein (LDL) [18]. HDL is formed from lipid-free apolipoprotein A1 (ApoA1) in a process involving the removal of free cholesterol from peripheral tissues and the subsequent esterification of some of the free cholesterol into cholesteryl esters via the actions of adenosine triphosphate binding cassette transporter A1 and lecithin:cholesterol acyltransferase (LCAT) [11]. The enrichment of HDL with triglycerides makes it a substrate for hepatic lipase (HL) which then hydrolyses the triglycerides, resulting in dissociation of the lipid-free ApoA1 and a decrease in size of the HDL particle, forming small-dense HDL [18].

## Materials and Methods

### Selection of the Candidate Gene for the Review

To identify candidate genes which have been reported by genome-wide association (GWA) studies to influence blood lipid levels, a literature search was undertaken in December 2020, using the following keywords: (genome-wide association study OR genome-wide association scan OR genome-wide association analysis OR GWAS OR GWA) AND (Lipids OR HDL OR LDL OR VLDL OR total cholesterol OR triglycerides OR triacylglycerol OR blood lipids). The results showed that, out of 32 identified studies (Supplemental Table 1), 20 GWA studies reported statistically significant associations between *CETP* and lipids [19–38], while *LPL* was reported by 18 GWA studies [19, 20, 22, 24, 25, 28, 29, 31–41] and *APOE* was reported by 10 GWA studies [22, 24, 25, 27, 29, 30, 32, 33, 34, 37]. *CETP* was then chosen for the review as it had the highest number of hits compared to *LPL* and *APOE*.

### Study identification

To identify published articles, a literature search was undertaken using PubMed (https://pubmed.ncbi.nlm.nih.gov/) and Google Scholar (https://scholar.google.com/). The search covered the earliest date of indexing through to July 2021. For PubMed, the following key terms were used: (CETP OR cholesteryl ester transfer protein) AND (polymorphism OR gene OR SNP OR single nucleotide polymorphism OR genetic variation OR genetic variant OR rs3764261 OR rs1532624 OR rs1800775 OR rs9989419 OR rs4783961 OR rs708272 OR rs7499892 OR rs2303790 OR rs16965220 OR rs247616 OR rs289708 OR rs12708980 OR rs247617 OR rs173539) AND (‘gene-diet interaction’ OR ‘diet-gene interaction’ OR ‘SNP-diet interaction’ OR ‘diet-SNP interaction’ OR ‘gene-nutrient interaction’ OR ‘nutrient-gene interaction’) AND (carbohydrate OR protein OR fat OR fibre OR sugar OR SFA OR MUFA OR PUFA OR Mediterranean diet OR Nordic diet OR B12 OR amino acids OR polyphenols OR egg intake OR caffeine intake OR green tea OR alcohol intake OR meat intake) AND (lipids OR HDL OR LDL OR VLDL OR total cholesterol OR triglycerides OR triacylglycerol OR blood lipids OR serum lipids). The key terms for Google Scholar were (CETP AND ‘gene-diet interaction’ AND lipids). Only studies published in English were included.

### Study Selection

**Fig. 2:**
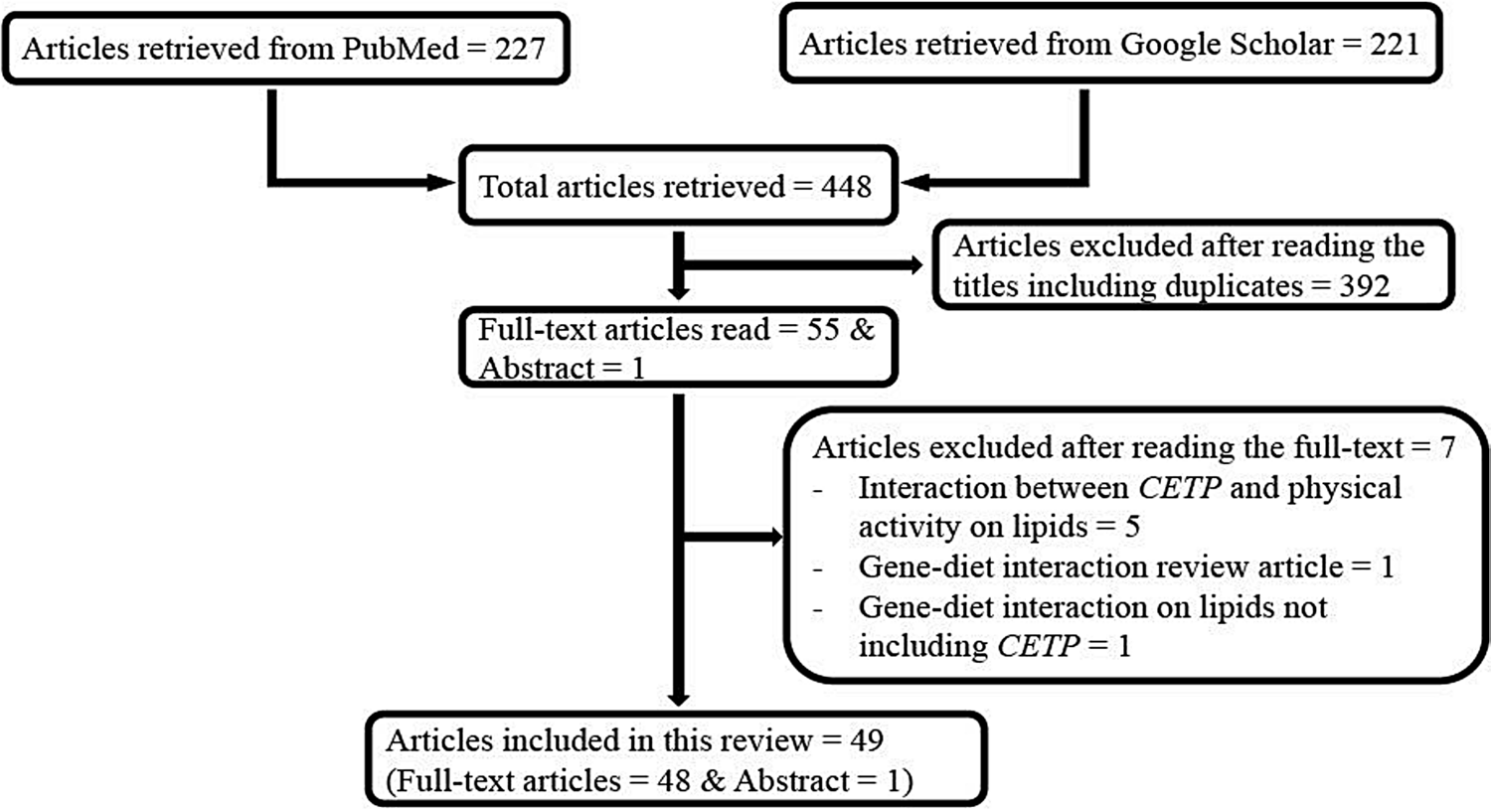
Flowchart of studies identified in the literature for *CETP*–diet interaction on lipids

The search strategies above yielded a total of 448 articles from the two databases (227 from PubMed and 221 from Google Scholar) as shown in Fig. 2. Titles of all the studies were first read to determine their relevance to the topic. Full-text of those found to be relevant were then read in detail to determine eligibility for inclusion. The criteria for inclusion in the review were as follows: gene–diet interaction studies involving *CETP* gene polymorphisms and blood lipids. Only studies conducted in humans were included and, after applying the inclusion and exclusion criteria, 49 articles were found to be eligible, of which one article was published as an abstract. The studies excluded after reading the full-text were those focusing on interaction between *CETP* and physical activity on lipids; gene–diet interaction on lipids not including *CETP*; and gene–diet interaction review articles. Full-text of 48 eligible studies was read in detail and the results were extracted for analysis (Supplemental Tables 2 and 3). The results of one study [42] which was published as an abstract were also extracted and included in the tables. The studies consisted of 28 observational studies (Supplemental Table 2) and 21 interventional studies (Supplemental Table 3).

### Data Extraction

The studies were identified by a single investigator and the following data were double-extracted independently by one reviewer: first author, publication year, location or ethnicity of participants, sample size, mean age, study design, reference SNP (rs) ID, genotype and minor allele. Corresponding authors were contacted to provide additional information where needed.

## Results of Database Search

**Fig. 3:**
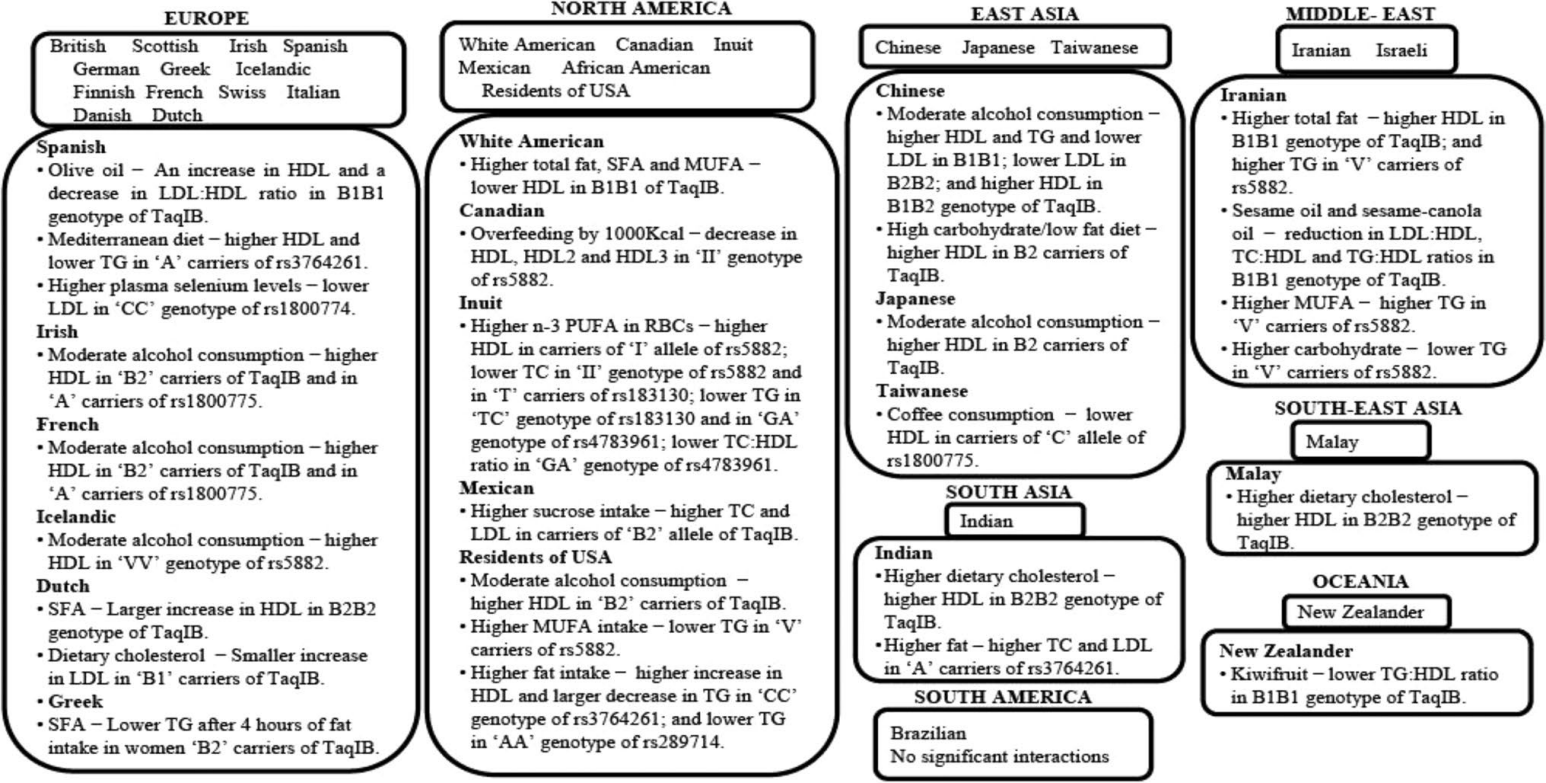
*CETP*–diet interaction studies and the interaction findings in multiple ethnicities

This section reviews studies examining the interaction between dietary factors and *CETP* SNPs on blood lipids. The rs708272 (TaqIB), the most widely studied *CETP* SNP, was investigated by 31 studies. The second most studied SNP was rs5882 (I405V), accounting for 16 studies. The *CETP* SNPs rs3764261 and rs1800775 were each examined by 6 studies. All the studies were conducted in adults except for one study which was carried out in prepubertal children [43]. The ethnicities covered by the studies included British, White American, Spanish, Mexican, Chinese and Iranian as shown in Fig. 3. A wide range of dietary factors were investigated by the 28 observational studies, and these included dietary carbohydrate, protein, saturated fatty acids (SFA), monounsaturated fatty acids (MUFA), polyunsaturated fatty acids (PUFA), coffee, sucrose, total energy intake and alcohol consumption. The 21 dietary intervention studies also focused on a variety of diets including Mediterranean diet, plant sterol ester, sesame oil, canola oil and rapeseed oil.

### TaqIB (SNP rs708272 G > A)

The major allele (‘G’) is also called the ‘B1’ allele while the minor allele (‘A’) is also referred to as the ‘B2’ allele. Eight out of seventeen observational studies reported a significant association between TaqIB genotypes, dietary factors and blood lipids. In a cross-sectional study involving 129 Iranian patients with type 2 diabetes (T2D) without dyslipidaemia [15], a higher intake of total fat (>34.9% from total energy intake) was associated with higher HDL in participants with ‘B1B1’ genotype (mean HDL (mg/dl) for high total fat intake (>34.9% from total energy) vs low total fat intake (≤ 34.9% from total energy) = 58.6 ± 4.1 vs 36.5 ± 6.5; *P*_interaction_ = 0.02). Those with ‘B2B2’ genotype who had a higher intake of total fat (>34.9% from total energy) also had higher HDL (mean HDL (mg/dl) for high total fat intake (>34.9% from total energy) vs low total fat intake (≤ 34.9% from total energy) = 59.0 ± 4.2 vs 55.8 ± 3.3) but the interaction was more pronounced in individuals with ‘B1B1’ genotype, while in those with ‘B1B2’ genotype, the interaction was not observed. A prospective cohort study of 603 men with T2D in the USA (96% of whom were white) [13] on the other hand reported that a higher intake of total fat (>33.5% from total energy intake), animal fat (>19.9% from total energy intake), SFA (>11.47% from total energy intake) and MUFA (>12.75% from total energy intake) was associated with lower HDL in participants with ‘B1B1’ genotype compared to those with ‘B2B2’ genotype (mean HDL (mg/dl) for low total fat intake (≤ 33.5% of energy) vs high total fat intake (>33.5% of energy): 40.0 ± 0.03 vs 36.2 ± 0.02 for ‘B1B1’, 41.5 ± 0.03 vs 44.9 ± 0.03 for ‘B2B2’, *P*_interaction_ = 0.003; mean HDL (mg/dl) for low animal fat intake (≤ 19.9% of energy) vs high animal fat intake (>19.9% of energy): 39.7 ± 0.02 vs 36.2 ± 0.03 for ‘B1B1’, 42.2 ± 0.04 vs 43.5 ± 0.03 for ‘B2B2’, *P*_interaction_ = 0.02; mean HDL (mg/dl) for low SFA intake (≤ 11.47% of energy) vs high SFA intake (>11.47% of energy): 39.8 ± 0.02 vs 36.2 ± 0.03 for ‘B1B1’, 42.2 ± 0.04 vs 43.8 ± 0.03 for ‘B2B2’, *P*_interaction_ = 0.02; mean HDL (mg/dl) for low MUFA intake (≤ 12.75% of energy) vs high MUFA intake (>12.75% of energy): 39.3 ± 0.03 vs 36.5 ± 0.02 for ‘B1B1’, 41.9 ± 0.03 vs 44.2 ± 0.03 for ‘B2B2’, *P*_interaction_ = 0.04). The difference in the findings might be due to the type of fat consumed since the Iranian study only considered total fat intake while the American study investigated types of fat. Furthermore, the variation in frequency of the TaqIB SNP might also contribute to the difference in the findings. In the Iranian study [15], only 8 out of 127 normolipidemic individuals had the ‘B1B1’ genotype, but in the American study [13] 192 out of 603 participants had the ‘B1B1’ genotype. Thus, while these two studies were both conducted in patients with T2D, there is a wide variation in frequency of the ‘B1B1’ genotype between the two studies and this affects the interpretation of the findings. In an animal study performed in feral adult male St. Kitts vervet monkeys (*Cercopithecus aethiops sabeus*) [44], SFA was shown to increase CETP activity, thereby reducing HDL levels which might explain the findings of the study in the American population [13]. However, in the animal study [44], the effect of SFA on CETP activity was only observed when cholesterol was added to the diet. SFA has also been shown to lower the number of LDL receptors in the liver, which slows the removal of apolipoprotein B (ApoB)–containing lipoproteins [45], with the resulting effect of a decrease in HDL levels. It has also been demonstrated that the effect of dietary fat on *CETP* expression is not dependent solely on the composition of fat, but also on the amount of fat [46], although the mechanisms under which total fat affects *CETP* expression are still unclear [15]. A cross-sectional study of 2858 Chinese participants, 761 Malay participants and 588 Asian Indian participants [17] demonstrated that participants with ‘B2B2’ genotype had a significantly higher increase in HDL in response to a higher intake of dietary cholesterol compared to those with ‘B1B1’ and ‘B1B2’ genotypes, but the interaction was only significant in Asian Indians (*P*_interaction_ = 0.0230) and Malays (*P*_interaction_ = 0.0460). A cross-sectional study of 215 Mexican-Mestizos [47] also showed that a higher sucrose intake (≥ 5% of total energy per day) was linked to increased levels of total cholesterol and LDL in individuals with ‘B1B2’/‘B2B2’ genotype compared to those with ‘B1B1’ genotype (mean total cholesterol (mg/dl) (95% confidence interval): 200.19 (184.79–215.60) vs 165.55(142.21–188.89), *P*_interaction_ = 0.0340; mean LDL (mg/dl) (95% confidence interval): 128.64 (113.59–143.69) vs 99.29 (75.52–123.05), *P*_interaction_ = 0.0370). As this study [47] was the only one which investigated sucrose intake, and considering that the sample size was 215, further studies are needed to corroborate these findings.

Several studies have investigated the interaction between alcohol intake and TaqIB genotype on HDL, LDL and TG [48–51]. In a cross-sectional study of 758 healthy Chinese participants [48], individuals with ‘B1B1’ genotype who consumed any amount of alcohol had higher HDL (mean HDL (mmol/l): 2.09 ± 0.46 vs 1.94 ± 0.38; *P*_interaction_ < 0.01), higher TG (mean TG (mmol/l): 1.42 ± 2.71 vs 0.94 ± 0.36; *P*_interaction_ < 0.05 ) and lower LDL (mean LDL (mmol/l): 2.24 ± 0.65 vs 2.65 ± 3.01; *P*_interaction_ < 0.01) compared to those with ‘B1B1’ genotype who did not drink alcohol. Those with ‘B1B2’ genotype who consumed any amount of alcohol also had higher HDL (mean HDL (mmol/l): 2.17 ± 0.55 vs 2.02 ± 0.50; *P*_interaction_<0.05) compared to individuals with ‘B1B2’ genotype who did not drink alcohol; and lower TG (mean TG (mmol/l): 1.01 ± 0.86 vs 1.42 ± 2.71; *P*_interaction_<0.05) compared to those with ‘B1B1’ who consumed any amount of alcohol. There were no significant interactions between alcohol intake and TG or HDL in participants with ‘B2B2’ genotype. This study also observed that ‘B2B2’ individuals who drank any amount of alcohol had lower LDL than ‘B2B2’ participants who did not drink alcohol (mean LDL in mmol/l: 2.20 ± 0.52 vs 2.41 ± 0.86; *P*_interaction_ < 0.0500), while there were no significant interactions between alcohol intake and LDL in those with ‘B1B2’ genotype. Similar findings were reported in a nested case-control study involving 505 patients with coronary heart disease (CHD) and 1010 healthy controls from different ethnicities in the US population [49] in which it was observed that, among healthy individuals, a higher intake of alcohol (≥ 15g/day) was linked to higher HDL in participants carrying the ‘B2’ allele compared to those with ‘B1B1’ genotype, with ‘B2B2’ individuals having the highest HDL (*P*_interaction_ < 0.0100). These findings are consistent with the results of a case-control study consisting of 608 Irish and French men with myocardial infarction (MI) and 742 healthy controls [51], which reported that, among individuals with a higher alcohol intake (≥ 75g/day), those carrying the ‘B2’ allele had higher mean plasma HDL (30% higher for ‘B2B2’ and 13% higher for ‘B1B2’) than those with the ‘B1B1’ genotype (*P*_interaction_ < 0.0001). Likewise, a cross-sectional study of 1729 Japanese participants [50] reported that, among women who consumed any amount of alcohol, those with ‘B2B2’ genotype had higher HDL than those with ‘B1B1’ or ‘B1B2’ genotype (mean HDL (mmol/l): 1.57 ± 0.03 for ‘B1B1’; 1.57 ± 0.03 for ‘B1B2’; 1.79 ± 0.06 for ‘B2B2’; *P*_interaction_ = 0.0220); while in men who consumed ≥ 2 drinks/day, those carrying the ‘B2’ allele had higher HDL than those with ‘B1B1’ genotype (mean HDL (mmol/l): 1.37 ± 0.03 for ‘B1B1’, 1.44 ± 0.03 for ‘B1B2’, 1.49 ± 0.05 for ‘B2B2’; *P*_interaction_ = 0.0490). These findings suggest that alcohol intake could alter lipid profiles by increasing HDL in both ‘B1’ and ‘B2’ carriers; however, the underlying mechanism is unclear and considering that alcohol intake has been linked to other health issues such as liver cirrhosis, the overall benefit needs to be carefully considered. Moreover, interaction between alcohol intake and TaqIB genotype on blood lipids has been investigated by 12 studies and eight of the studies have not found significant interactions [13, 15, 52–57].

Six out of fourteen dietary intervention studies found significant interactions between TaqIB genotype, dietary factors and blood lipids. Three of the interactions were observed in participants carrying the ‘B2’ allele while the remaining three were reported in those with the ‘B1B1’ genotype. A 6-day dietary intervention study [58], using high carbohydrate/low-fat diet in 56 healthy Chinese individuals, showed that those carrying the ‘B2’ allele had higher HDL concentrations (mean HDL (mg/dl): 56.14 ± 10.69 after washout diet vs 59.77 ± 10.62 after high carbohydrate/low-fat diet; *P*_interaction_ < 0.0500) but the interaction was not observed in individuals with ‘B1B1’ genotype. As the duration of this intervention was only 6 days, intervention studies with longer duration are required to confirm the effect of carbohydrate on HDL in individuals carrying the ‘B2’ allele. In a meta-analysis of 26 dietary interventions using SFA, trans fat, dietary cholesterol and the coffee diterpene cafestol in 405 healthy Dutch participants over a 20-year period [59], participants with ‘B2B2’ genotype had a larger increase in HDL in response to SFA compared to those with ‘B1B1’ or ‘B1B2’ genotypes (mean change in HDL (mmol/l): 0.08 ± 0.02 for ‘B2B2’, 0.03 ± 0.01 for ‘B1B2’, 0.04 ± 0.02 for ‘B1B1’ genotype; *P* = 0.0400), while participants carrying the ‘B1’ allele had a smaller increase in LDL in response to dietary cholesterol than those with the ‘B2B2’ genotype (mean change in LDL (mmol/l): 0.27 ± 0.14 for ‘B1B1’, 0.35 ± 0.08 for ‘B1B2’, 0.75 ± 0.15 for ‘B2B2’; ‘B1B1’ vs ‘B2B2’, *P* = 0.0300; ‘B1B2’ vs ‘B2B2’, *P* = 0.0100). In an oral fat tolerance test performed in 80 Greek participants who were heterozygous for familial hypercholesterolemia (HFH) and 11 control participants [60], it was demonstrated that, among participants in the HFH group who showed an abnormal postprandial TG response (TG concentration of >220 mg/dl), men with the ‘B2’ allele had higher levels of TG than women with the ‘B2’ allele after 4 hours of fat intake (279 ± 95 vs 239 ± 65 mg/dl, *P* = 0.0300) but there were no reports of significant interactions in participants with ‘B1B1’ genotype.

Statistically significant interactions between carriers of the ‘B1’ allele and dietary factors were reported by three dietary intervention studies [43, 61, 62]. In a randomised triple-blind crossover trial performed in 95 Iranian patients with T2D and 73 healthy controls using three diets: sesame oil, canola oil and sesame-canola oil [61], it was demonstrated that, in the T2D group, those with ‘B1B1’ genotype had a significant reduction in lipid ratios after consuming sesame oil and sesame-canola oil (change in LDL:HDL (mg/dl): −1.29, *P*_interaction_ = 0.0270; change in TC:HDL (mg/dl): −2.82, *P*_interaction_ = 0.0240; and change in TG:HDL (mg/dl): −7.00, *P*_interaction_ = 0.0250) but there were no reports of significant reductions in lipid ratios in participants carrying the ‘B2’ allele. Another randomised controlled trial (RCT) performed in 85 New Zealander men with hypercholesterolemia, involving a 4-week healthy diet vs healthy diet plus two kiwi fruits per day [62], also showed that, among participants with ‘B1B1’ genotype, consumption of kiwi fruit resulted in lower TG:HDL ratio than the control diet (mean change in TG:HDL (mmol/l): −0.14 ± 0.51 for kiwifruit vs 0.09 ± 0.56 for control diet, *P* = 0.03; *P*_interaction_ < 0.05), while in individuals carrying the ‘B2’ allele, the interaction was not observed. Similar results were also observed in a crossover intervention conducted in Spanish prepubertal children with mild hypercholesterolemia, consisting of consumption of cow’s skim milk vs cow’s skim milk enriched with virgin olive oil for two periods of 6 weeks [43]. It was observed that intake of olive oil–enriched skim milk resulted in a larger increase in HDL and a decrease in LDL:HDL ratio in participants with ‘B1B1’ genotype compared to those carrying the ‘B2’ allele (mean change in HDL (mmol/l) (95% confidence interval): 0.179 (0.096 to 0.262) for ‘B1B1’ vs 0.089 (0.032 to 0.146) for carriers of ‘B2’, *P*_interaction_ < 0.0010; mean change in LDL:HDL ratio (mmol/l) (95% confidence interval): −0.470 (−0.729 to 0.211) for ‘B1B1’ vs −0.097 (−0.275 to 0.081) for carriers of ‘B2’, *P*_interaction_ < 0.0010). While these studies show that individuals with the ‘B1B1’ genotype could benefit from consuming these diets, the interactions were reported only in those with either T2D [61] or hypercholesterolemia [43, 62] indicating that these results may not apply to healthy participants and hence, this limits the wider application of the findings.

The TaqIB, located in intron 1 of the *CETP* gene, is considered to be non-functional and is believed to serve as a marker for functional SNPs in the promoter region [13, 17, 63]. The ‘B1’ allele differs from the ‘B2’ allele by the presence of a restriction site for TaqI endonuclease [17]. The ‘B1’ allele is believed to be associated with higher CETP activity, resulting in lower HDL and higher serum TG, and is considered a risk factor for dyslipidaemia [47]. This is supported by some of the studies as participants with the ‘B1B1’ genotype tended to have lower HDL [15, 17, 49, 56]. Nonetheless, the results suggest that people with this genotype can increase their HDL and modify their genetic risk by consuming sesame oil, canola oil, olive oil and kiwi fruit among others, although larger studies covering different ethnicities are warranted to tailor nutritional advice based on ethnicity and genetic profile.

### SNP rs5882 (I405V G > A)

The SNP rs5882 (I405V) results in a substitution of valine (V) for isoleucine (I); hence, the ‘G’ allele is also called the ‘V’ allele while the ‘A’ allele is also known as the ‘I’ allele. The frequency of the ‘V’ allele is 34% globally but in Africans it is 58%, while in Asians it is 48% and in Europeans it is 32% [64]. Six out of eight observational studies found statistically significant interactions between this SNP and dietary factors on blood lipids. A cross-sectional analysis of 101 individuals from different ethnicities in the US population [10•] showed that a higher MUFA intake (>31g/day) was associated with lower TG in participants carrying the minor allele (‘V’) (*P*_interaction_ = 0.0060) but there were no reports of significant interactions in individuals with ‘II’ genotype. A longitudinal study of 4700 Iranian participants over 3.6 years [65] reported that a higher MUFA intake was linked to increased levels of TG in participants carrying the ‘V’ allele (mean changes in TG (mg/dl) across quartiles of MUFA intake: −3.03, 1.73, 8.06, 8.85; *P*_interaction_ = 0.0010), but the interaction was not observed in those with ‘II’ genotype. This study also observed that a higher intake of total fat correlated with increased levels of TG in those carrying the ‘V’ allele (mean changes in TG (mg/dl) across quartiles of total fat intake: −1.90, 2.6, 6.06, 8.88; *P*_interaction_ = 0.0010) but the interaction was not significant in those with ‘II’ genotype. A higher carbohydrate intake was also found to be associated with decreased levels of TG in ‘V’ allele carriers (mean changes in TG (mg/dl) across quartiles of carbohydrate intake: 6.65, 7.29, 4.42, −3.28; *P*_interaction_ = 0.0100) but the interaction was not significant in individuals with ‘II’ genotype [65]. Interactions with MUFA were also reported in a nested case-control of 441 Iranian participants with metabolic syndrome and 844 healthy controls [66] wherein carriers of the ‘V’ allele had a reduced risk of low HDL with a low intake of MUFA (<8.4% of energy) and an increased risk of low HDL with a higher intake of MUFA (9.6–11% of total energy intake) compared to those with ‘II’ genotype (odds ratio for low HDL across quartiles of MUFA intake: 0.49, 0.66, 0.88, 0.66 for carriers of ‘V’ allele vs 1, 0.61, 0.62, 0.68 for ‘II’ genotype; *P*_interaction_ = 0.0200). The findings of these studies suggest that the SNP rs5882 (I405V) may modify the link between fat intake and blood lipids. A higher intake of MUFA and total fat appears to be unfavourable in Iranian participants carrying the ‘V’ allele by leading to an increase in TG levels and the risk of low HDL while carbohydrate intake seems to be beneficial in reducing TG levels in these ‘V’ allele carriers [65, 66]. Conversely, the study in the US population [10•] implies MUFA is beneficial in individuals carrying the ‘V’ allele. As this study [10•] was performed in participants from different ethnicities, it is difficult to confirm ethnicity as a reason for the differential response to MUFA. Moreover, the study was performed in participants with overweight and obesity which could influence the findings since obesity is known to alter the interaction between diet and genotype on lipids [10•]. Nonetheless, the Iranian case-control study [66] also involved participants with metabolic syndrome as well as healthy controls; but, the study did not report the findings for healthy controls. It has been demonstrated that *CETP* transgenic mice fed with MUFA had improved LDL receptor activity with a corresponding increase in the uptake of ApoB-containing lipoproteins by the liver [67], which could explain the reduction in TG levels associated with a high MUFA diet. The lipid-lowering effect of MUFA has also been linked to a decrease in expression of the transcription factor liver X receptor α (LXRα) which is involved in CETP activation [68]. Moreover, it has been argued that animal-based sources of MUFA also contain substantial amounts of SFA which could mask the effects of MUFA [69], implying that the source of MUFA needs to be taken into account when assessing the impact of MUFA on lipid-related outcomes.

In a cross-sectional study of Icelandic participants (152 men and 166 women) [70], alcohol intake was found to be associated with higher HDL in men with ‘VV’ genotype (13.7% higher HDL than ‘II’ genotype) compared to men with ‘II’ or ‘IV’ genotype (*P*_interaction_ < 0.0200) but the interaction was not statistically significant in women. Interactions with HDL were also observed in a cross-sectional study of 553 Inuit participants [71] in which higher levels of omega 3 polyunsaturated fatty acids (n-3 PUFA) in red blood cells (RBCs) was associated with higher HDL in participants carrying the major allele (‘I’) compared to those with ‘VV’ genotype (*β* (mmol/l) = 0.0263 ± 0.0115 for ‘IV’ genotype, *β* (mmol/l) = 0.0017 ± 0.0131 for ‘II’ genotype; *P*_interaction_ = 0.0271). The study also found that n-3 PUFA in RBCs had a negative correlation with total cholesterol in participants with ‘II’ genotype compared to those with ‘VV’ or ‘IV’ genotype (*β* (mmol/l) = −0.0290 ± 0.0307; *P*_interaction_ = 0.0334). In another cross-sectional study of 553 Inuit participants [16], individuals with ‘II’ genotype had a greater increase in total cholesterol with a higher intake of total fat than those with ‘VV’ or ‘IV’ genotype (*β* (mmol/l) = 0·0024 ± 0·0026; *P*_interaction_ = 0.0460). These findings imply that while n-3 PUFA intake was beneficial for Inuit participants carrying the ‘I’ allele [71], higher total fat intake was not favourable for these participants [16]. PUFA is believed to promote the synthesis of LDL receptors which has the effect of increasing hepatic uptake of ApoB-containing lipoproteins [72], thereby raising the levels of HDL. To understand how PUFA affects regulation of the *CETP* gene, a study [73] was conducted in *CETP* transgenic mice which demonstrated that n-3 PUFA resulted in elevated *CETP* messenger RNA (mRNA) and protein levels, possibly by being a ligand for peroxisome proliferator–activated receptors α (PPARα), which is involved in the regulation of lipid-related genes [73]. However, increased *CETP* activity is known to have an inverse effect on HDL levels and does not explain the beneficial effect on HDL observed in the Inuit study. This raises the question of whether particular *CETP* SNPs dictate the response of the CETP protein to n-3 PUFA.

Only one of the six dietary intervention studies reported significant interactions between the I405V SNP and dietary factors on blood lipids. In this study [74], Canadian monozygotic twins (12 pairs) who were overfed by 1000 kcal per day for a period of 100 days showed a significant decrease in HDL, HDL_2_ and HDL_3_ in those with ‘II’ genotype compared to individuals with ‘VV’ genotype (mean change in HDL (mmol/l): −0.12 ± 0.04 vs 0.02 ± 0.04, *P* = 0.02; mean change in HDL_2_ (mmol/l): −0.08 ± 0.03 vs 0.03 ± 0.03, *P* = 0.04; mean change in HDL_3_ (mmol/l): −0.04 ± 0.02 vs −0.004 ± 0.02, *P* = 0.0020), but there were no reports of significant interactions in individuals with ‘IV’ genotype. The ‘II’ genotype of SNP rs5882 is believed to affect the ability of the CETP protein to mediate the exchange of cholesteryl esters for TG, resulting in increased TG concentrations [66], although this SNP has not been reported by any of the 32 GWASs to impact on lipids.

It has also been shown that the ‘VV’ genotype of the SNP rs5882 is associated with lower plasma CETP levels and increased HDL concentration [75]; however, baseline HDL data for participants with the ‘VV’ genotype was not available for all the studies because there were not enough participants with the ‘VV’ genotype in the two Iranian studies [65, 66]. Also, in the two Inuit studies [16, 71], baseline HDL data was not recorded separately for ‘VV’, ‘IV’ or ‘II’ genotype. However, in the Icelandic study [70], those with the ‘VV’ genotype had higher baseline HDL levels. Overall, the findings indicate that the SNP rs5882 may modify dietary response to lipids, but further studies are needed to clarify the differences in the results of some of the studies.

### SNP rs3764261 (C > A)

Significant interactions between dietary factors and SNP rs3764261 on blood lipids were observed in two out of four observational studies. In a longitudinal study of 4700 Iranian participants over 3.6 years [65], it was reported that a higher fish intake was associated with a larger decrease in total cholesterol (TC) in participants carrying the minor allele (‘A’) (mean changes in TC (mg/dl) with quartiles of fish intake: 8.02, 6.93, 6.54, 5.58) compared to those carrying two copies of the major allele (‘C’) (mean changes in TC (mg/dl) with quartiles of fish intake: 3.65, 6.62, 4.57, 8.93) (*P*_interaction_ = 0.02). Interactions with fat intake were also observed in a cross-sectional study of 3342 Indian participants [76] in which a high dietary fat intake (≥ 76.98g/day) was associated with increased levels of TC (*β* (mmol/l) = 0.097 ± 0.041; *P*_interaction_ = 0.018) and LDL (*β* (mmol/l) = 0.085 ± 0.041; *P*_interaction_ = 0.0420) in participants carrying the ‘A’ allele but there were no reports of interactions in those with ‘CC’ genotype. A high-fat diet has been demonstrated to increase CETP activity in transgenic mice [77] which has the effect of increasing TC and LDL and could account for the findings reported. Moreover, the SNP rs3764261 (C > A) is located in the 5^1^ region of the *CETP* gene and has been shown to regulate expression of the gene, the ‘C’ allele being associated with increased *CETP* expression and reduced HDL levels [78].

Two dietary intervention studies reported statistically significant interactions between the SNP rs3764261 and dietary factors on blood lipids. In a prospective, randomized, single-blind controlled dietary intervention trial carried out in 424 Spanish patients who had acute coronary syndrome (ACS) or CHD and also had metabolic syndrome [14], wherein participants consumed either a Mediterranean diet or a low-fat diet, it was observed that, after 1 year, consumption of Mediterranean diet was associated with higher HDL and lower TG in participants carrying the ‘A’ allele compared to those with ‘CC’ genotype (mean HDL (mg/dl): 41 vs 38, *P*_interaction_ = 0.0060; mean TG (mg/dl): 130 vs 146, *P*_interaction_ = 0.0400). This finding indicates that Mediterranean diet might be beneficial in increasing HDL in Spanish participants with ACS or CHD who carry the ‘A’ allele [14]; however, this finding might not be applicable to healthy individuals. In another study which was performed on participants from different ethnicities and involved a 2-year randomised weight loss trial, consisting of low-fat diet (20% fat) vs high-fat diet (40% fat), and a 2-year RCT consisting of low-fat diet (30% fat) vs low carbohydrate (high-fat) diet [46], the combined results of the two interventions showed that, among participants with ‘CC’ genotype, those in the high-fat diet group had a higher increase in HDL (11.7 vs 4.5%; *P*_interaction_ = 0.01) and a larger decrease in TG (−25.1 vs −11.7%; *P*_interaction_ = 0.0007) than those in the low-fat diet group, but there were no significant interactions in participants with ‘CA’ or ‘AA’ genotype. These results suggest that a high-fat diet (40% fat) in individuals from different ethnicities who have the ‘CC’ genotype might contribute to increased HDL and reduced TG levels [46] although the findings are not in agreement with the study performed in *CETP* transgenic mice [77] in which a high-fat diet resulted in increased CETP activity which lowered HDL levels. Considering that this was a weight loss intervention, it is unclear whether the changes in lipid levels were due to the high-fat diet or the loss of weight or both since physical activity has been shown to interact with genetic risk score and impact on waist-hip ratio [79]. Moreover, the ‘C’ allele of the SNP rs3764261 is regarded as a significant risk factor for low HDL [78, 80] although it has been demonstrated that this risk can be overcome by weight gain prevention [78]. The SNP rs3764261 has also been shown by GWASs to influence HDL levels in Asian Indians [19, 28], Japanese [26, 31], African-American [29], Chinese [20], Lebanese [21] and Finnish [35] but the evidence indicates that this SNP has not been extensively studied by gene–diet interaction studies. Therefore, further studies in different ethnicities are required to confirm the effect of the SNP in modifying dietary response to lipids.

### C-629A (SNP rs1800775 C > A)

The SNP rs1800775 has been shown to be associated with HDL in seven of the nineteen GWASs [19, 22, 24, 31, 34, 35, 37]. Two out of five observational studies reported significant interactions between dietary factors and the SNP rs1800775 (C-629A) on blood lipids. In a cross-sectional study of 9075 Taiwanese participants [81], consumption of coffee was found to be associated with lower HDL in women carrying the minor allele (‘C’) compared to women with ‘AA’ genotype (*β* (mg/dl) = −1.8095 for ‘AC’ genotype, *β* (mg/dl) = −2.8151 for ‘CC’ genotype; *P*_interaction_<0.0001), and in men carrying the ‘C’ allele compared to men with the ‘AA’ genotype (*β* (mg/dl) = −1.9623 for ‘AC’ genotype, *β* (mg/dl) = −2.7154 for ‘CC’ genotype; *P*_interaction_<0.0001). A case-control study consisting of 568 Irish and French men with MI and 668 healthy controls [82] showed that, among individuals carrying the major allele (‘A’), alcohol consumption was associated with higher HDL in healthy participants (*P*_interaction_<0.0020) and in patients who were not treated with lipid-lowering medication (*P*_interaction_<0.0010), while in individuals with ‘CC’ genotype, there was no association between alcohol intake and HDL. The results suggest that dietary factors other than fat intake may also play a role in modulating lipid levels, but these interactions need to be explored further to allow for comparison of results across multiple ethnic groups. The SNP rs1800775 (C-629A) is located in the promoter region of the *CETP* gene and the ‘A’ allele is associated with reduced *CETP* expression and higher HDL levels [17]. The ‘A’ allele of SNP rs1800775 (C-629A) is in a high degree of linkage disequilibrium with the ‘B2’ allele of SNP TaqIB and it is believed that this association is responsible for the protective effect of the ‘B2’ allele [17]. However, some are of the view that there might be other functional SNPs that are in linkage disequilibrium with TaqIB apart from SNP rs1800775 (C-629A) but it is unclear what these SNPs are [13, 63]. Moreover, despite the SNP rs1800775 (C-629A) being reported by several GWASs to be associated with blood lipids, this SNP has not been extensively studied in gene–diet interaction studies. To date, only one dietary intervention study [83] investigated the SNP rs1800775 (C-629A) which also failed to demonstrate any significant SNP–diet interactions on lipids. This study was an RCT performed in 490 participants from different ethnicities in the UK population and involved a reference diet (∼18% SFA, 12% MUFA, 38% total fat, 45% carbohydrate (CHO)) for 4 weeks, followed by 1 of three diets: a MUFA diet (∼10% SFA, 20% MUFA, 38% total fat, 45% CHO); a low-fat diet (∼10% SFA, 11% MUFA, 28% total fat, 55% CHO); or the reference diet for 24 weeks. The findings overall indicate that further large studies are needed to confirm the effect of the SNP rs1800775 in altering lipid profiles in response to diet.

### Other SNPs

Other *CETP* SNPs which have been reported to interact with dietary factors and influence blood lipids are SNPs rs183130 (C-4502T), rs4783961 (G-971A), rs289714 (C>T) and rs1800774 (C > A). In the cross-sectional study of 553 Inuit participants [84], higher levels of n-3 PUFA in RBCs was linked to lower TC levels in participants carrying the minor allele (‘T’) of the SNP rs183130 (C-4502T) compared to those with ‘CC’ genotype (*β* (mmol/l) = −0.0632 ± 0.0241 for ‘CT’ genotype, *β* (mmol/l) = −0.0421 ± 0.0343 for ‘TT’ genotype; *P*_interaction_ = 0.0326); and lower TG levels in those with the ‘TC’ genotype of the SNP rs183130 (C-4502T) compared to individuals with ‘TT’ genotype (*β* (mmol/l) = −0.0095 ± 0.0051 vs *β* (mmol/l) = 0.0073 ± 0.0073; *P*_interaction_ = 0.0300), while there were no reports of significant interactions between n-3 PUFA in RBCs and TG in participants with ‘CC’ genotype. This study also reported that individuals with the ‘GA’ genotype of the SNP rs4783961 (G-971A) who had higher levels of n-3 PUFA in RBCs had lower TG levels (*β* (mmol/l) = −0.0106 ± 0.0057; *P*_interaction_ = 0.0032) and lower TC:HDL ratio (*β* (mmol/l) = −0.0055 ± 0.0033; *P*_interaction_ = 0.0483) compared to participants with 2 copies of the minor allele (‘A’) of the SNP rs4783961 (G-971A). These findings point to a beneficial role of PUFA in Inuit participants carrying the ‘T’ allele of the SNP rs183130(C-4502T) and the ‘G’ allele of the SNP rs4783961 (G-971A). PUFA is believed to improve the breakdown of ApoB-containing particles, thereby reducing TG concentrations [66], which is consistent with this finding. However, in a cross-sectional study of 821 participants who were normal glucose tolerant and 861 participants with T2D, involving the transcription factor 7-like 2 (*TCF7L2*) gene [85], higher PUFA intake (mean PUFA intake of 29g/day) was linked to 1.64 mg/dl lower HDL while lower PUFA intake (mean PUFA intake of 9g/day) was associated with 1.96 mg/dl higher HDL in Asian Indian participants carrying the ‘T’ allele of the *TCF7L2* SNP rs12255372 compared to those with the ‘GG’ genotype (*P*_interaction_<0.0001 ). In another cross-sectional study involving 101 participants of different ethnicities in the US population [10•], it was reported that, among participants with two copies of the major allele (‘A’) of the SNP rs289714, those who consumed >92 g of total fat per day had lower TG levels (103 ± 63 mg/dl) than those who consumed <31 g of total fat per day (135 ± 15 mg/dl) (*P*_interaction_ = 0.0010). The interaction was significant for both the dominant and recessive modes of inheritance (*P*_interaction_ = 0.0010 and *P*_interaction_ = 0.0230 respectively), but there were no reports of significant interactions in individuals with ‘GG’ genotype. In another cross-sectional study of 1315 Spanish participants [86], higher plasma selenium levels were found to be associated with elevated LDL levels in all the three genotypes of the SNP rs1800774 but participants with two copies of the major allele (‘C’) had lower LDL compared to those with ‘CT’ and ‘TT’ genotypes (odds ratio per an interquartile range increase in plasma selenium (95% confidence interval): 0.97 (0.74 to 1.27) for ‘CC’, 1.76 (1.38 to 2.25) for ‘CT’, 3.20 (1.93 to 5.28) for ‘TT’ genotype; *P*_interaction_ = 0.0002). Selenium was also reported to be associated with lipid levels in a systematic review and meta-analysis [87], but it was shown to be linked to significant improvement in the levels of TC and TG and had no significant effect on LDL levels. A systematic review published in 2017, in which the results of 23 gene–diet interaction studies involving *CETP* were analysed [88], concluded that SNPs in the *CETP* gene may influence the effect of dietary factors on metabolic traits but that the findings from these studies were inconsistent and suggest that multiple factors might be involved.

## Conclusion

In summary, this review has identified statistically significant interactions between 17 dietary factors and 8 SNPs in the *CETP* gene on blood lipids in the following populations: Mexican, Iranian, Spanish, White American, Chinese, Malay, Indian, Irish, French, Japanese, New Zealander, Dutch, Greek, Icelandic, Inuit, Canadian, Taiwanese and residents of the USA. The SNPs showing significant interactions with dietary factors (such as total fat intake, MUFA, n-3 PUFA, Mediterranean diet, olive oil and sesame-canola oil) were TaqIB (rs708272 G > A); rs5882 (I405V); rs3764261 (C > A); rs1800775 (C-629A); rs183130(C-4502T); rs4783961 (G-971A); rs289714 (C>T) and rs1800774 (C > A). The macronutrient investigated by majority of the studies was dietary fat, comprising of total fat, SFA, MUFA and PUFA. Total fat intake accounted for majority of the interactions across different SNPs, being associated with unfavourable lipid outcomes in some individuals but not others.

Studies reporting significant interactions in individuals with the B1B1 genotype of the SNP TaqIB (rs708272) have been performed in participants with either T2D or hypercholesterolemia. Similarly, those reporting significant interactions in individuals carrying the ‘V’ allele of the SNP rs5882 have been conducted in participants with overweight and obesity or metabolic syndrome. Moreover, some of the significant interactions involving the SNP rs3764261 have also been reported in patients with ACS or CHD, suggesting that some of the findings of these studies may not apply to healthy participants. Overall, the findings suggest that *CETP* SNPs might alter blood lipid profiles by modifying responses to diet, but further large studies in multiple ethnic groups are warranted to identify individuals at risk of adverse lipid response to diet which is essential in developing dietary guidelines that are tailored to specific groups of people. Information on the underlying genetic factors for dyslipidaemia will also contribute to improved understanding of the mechanisms involved, which is central to the development of effective preventative strategies as well as identifying areas for further research.

## Supplementary Information

ESM 1 (DOCX 341 kb)
