# Supplementary material for: A Nutrigenetic Update on CETP Gene–Diet Interactions on Lipid-Related Outcomes
Source: Curr Atheroscler Rep. 2022 Jan 31;24(2):119–32. doi: 10.1007/s11883-022-00987-y (PMC8924099; doi:10.1007/s11883-022-00987-y)
Supplement: Supplementary file 1 — (DOCX 341 kb) [file 11883_2022_987_MOESM1_ESM.docx]

Supplemental Table 1. Lipid-related Genes Identified by Genome-wide Association Studies

| **Gene & SNP** | **Lipid Trait & P-value**^ab^ | | | | **Population & Sample Size** | **Age Group** |
| --- | --- | --- | --- | --- | --- | --- |
|  | **TC** | **HDL** | **LDL** | **TG** |  |  |
| **Khushdeep et al. (2019)** [[19](#_ENREF_19)] | | | | | | |
| *CELSR2*  rs646776 | 6.19×10^-3^ |  |  |  | Northern India, healthy individuals  n = 1036 | 45 – 60 (Men)  44 – 60 (Women) |
| *CELSR2*  rs646776 |  |  | 4.59×10^−3^ |  |  |  |
| *CETP*  rs3764261 |  | 6.291×10^-12^ |  |  |  |  |
| *CETP*  rs1532624 |  | 5.279×10^-9^ |  |  |  |  |
| *CETP*  rs1800775 |  | 3.968×10^-10^ |  |  |  |  |
| *LPL*  rs10096633 |  | 0.1 |  |  |  |  |
| *LPL*  rs12678919 |  | 0.2 |  |  |  |  |
| *CETP*  rs9989419 |  | 1.35×10^-3^ |  |  |  |  |
| *LPL*  rs4128744 |  | 0.03 |  |  |  |  |
| *CETP*  rs4783961 |  | 1.32×10^-4^ |  |  |  |  |
| *ZNF259*  rs964184 |  |  |  | 2.99×10^-6^ |  |  |
| *BUD13*  rs7350481 |  |  |  | 6.03×10^-7^ |  |  |
| *ZNF259*  rs6589567 |  |  |  | 1.73×10^-4^ |  |  |
| *LPL*  rs10096633 |  |  |  | 1.09×10^-6^ |  |  |
| *LPL*  rs4128744 |  |  |  | 9.78×10^-6^ |  |  |
| *QKI*  rs9458854 | 0.01 |  |  |  |  |  |
| *QKI*  rs9458855 | 0.01 |  |  |  |  |  |
| *REEP3*  rs7083226 | 9.42×10^-4^ |  |  |  |  |  |
| *REEP3*  rs7083226 |  |  | 0.01 |  |  |  |
| *TMCC2*  rs2290265 |  |  | 0.01 |  |  |  |
| *FAM129C*  rs4544358 |  | 0.28 |  |  |  |  |
| *FAM241B*  rs12771265 |  |  |  | 4.73×10^-6^ |  |  |
| *FAM241B*  rs4746882 |  |  |  | 2.71×10^-5^ |  |  |
| *LOC100506207* rs9393071 |  |  |  | 1.01×10^-5^ |  |  |
| **Zhou et al. (2013)** [[20](#_ENREF_20)] | | | | | | |
| *DOCK7*  rs11207995 | 3.27×10^-9^ |  |  |  | Han Chinese Healthy individuals  n = 3,451 | 63.0 ± 8.1  (Cohort 1)  37.5 ± 11.1 (Cohort 2) |
| *HMGCR*  rs10045497 | 3.80×10^-6^ |  |  |  |  |  |
| *HMGCR*  rs10045497 |  |  | 4.93×10^-7^ |  |  |  |
| *LPL*  rs328 |  |  |  | 1.91×10^-8^ |  |  |
| *LPL*  rs328 |  | 9.75×10^-12^ |  |  |  |  |
| *ABO*  rs507666 | 2.91×10^-7^ |  |  |  |  |  |
| *ABO*  rs507666 |  |  | 9.25×10^-8^ |  |  |  |
| *APOA1/C3/A4/A5* rs651821 |  |  |  | 1.35×10^-28^ |  |  |
| *LIPC*  rs2043085 |  | 3.02×10^-7^ |  |  |  |  |
| *TOMM40*  rs1160985 |  |  | 6.13×10^-6^ |  |  |  |
| *CETP*  rs3764261 |  | 6.65×10^-12^ |  |  |  |  |
| **Tekola-Ayele et al. (2015)** [[89](#_ENREF_89)] | | | | | | |
| *KSR2*  rs11610896 |  |  |  | 1.70×10^-5^ | African (Ghana and Nigeria)  n = 1427 | 45.73 ± 16.02 |
| *KSR2*  rs7964157 |  |  |  | 9.92×10^-6^ |  |  |
| *KSR2*  rs11610896 |  |  |  | 1.14×10^-5^ |  |  |
| *KSR2*  rs7964157 |  |  |  | 2.06×10^-5^ |  |  |
| *EDEM1-GRM7* rs1377212 |  | 7.20×10^-6^ |  |  |  |  |
| *EDEM1-GRM7* rs1377212 |  | 1.54×10^-5^ |  |  |  |  |
| *EDEM1-GRM7* rs1377212 |  | 1.71×10^-5^ |  |  |  |  |
| *EDEM1-GRM7* rs116357511 |  | 2.16×10^-5^ |  |  |  |  |
| *EDEM1-GRM7* rs116357511 |  | 2.11×10^-5^ |  |  |  |  |
| **Deek et al. (2019)** [[21](#_ENREF_21)] | | | | | | |
| Unknown  rs4288204 |  | 2.29×10^-6^ |  |  | Lebanese patients  Patients undergoing cardiac catherisation: n = 7,710  T2D: n = 775 | 62.32 ± 11.01 |
| ***RORA***  rs2062091 |  | 6.06×10^-6^ |  |  |  |  |
| ***CETP***  rs3764261 |  | 6.70×10^-7^ |  |  |  |  |
| ***CYP2B6***  rs17799912 |  | 5.41×10^-7^ |  |  |  |  |
| **Graff et al. (2017)** [[90](#_ENREF_90)] | | | | | | |
| ***CD86***  rs114378860 | 6.62×10^-9^ |  |  |  | Hispanic/Latino  TC: n = 12,731  TG & HDL: n=12,730  LDL: n = 12,467 | 18 - 74 |
| ***DNAH5***  rs183336356 | 2.80×10^-7^ |  |  |  |  |  |
| ***RP1-39 J2.1; SMOC2***  rs77635931 |  |  |  | 2.54×10^-7^ |  |  |
| ***SYNE1***  rs78768981 |  | 9.29×10^-9^ |  |  |  |  |
| ***DAGLB/GRID2IP*** rs77071750 |  | 1.01×10^-7^ |  |  |  |  |
| ***AUTS2***  rs191891263 |  | 2.06×10^-11^ |  |  |  |  |
| ***APOC3***  rs184637772 |  | 2.57×10^-6^ |  |  |  |  |
| ***DNAL1***  rs149886784 |  |  | 1.05×10^-8^ |  |  |  |
| **Hebbar et al. (2018**) [[91](#_ENREF_91)] | | | | | | |
| ***RPS6KA1***  **rs1002487** |  |  |  | 7.17×10^-11^ | Arab (Kuwait)  Healthy and Diabetic individuals  n = 1,913 | 46.77 ± 13.79 |
| ***LAD1***  **rs11805972** |  |  |  | 8.55×10^-11^ |  |  |
| ***OR5V1***  **rs7761746** |  |  |  | 1.89×10^-9^ |  |  |
| ***CTTNBP2, LSM8***  **rs39745** |  |  |  | 3.63×10^-9^ |  |  |
| ***PGAP3***  **rs2934952** |  |  |  | 3.17×10^-9^ |  |  |
| ***RP11-191L9.4,***  ***CERK***  **rs9626773** |  |  |  | 1.42×10^-9^ |  |  |
| ***ST6GALNAC5*** rs10873925 |  |  |  | 4.11×10^-8^ |  |  |
| ***SPP2, ARL4C*** rs4663379 |  |  |  | 8.38×10^-9^ |  |  |
| ***NPY1R***  rs10033119 |  |  |  | 8.79×10^-9^ |  |  |
| ***LINC00911, FLRT2***  rs17709449 |  |  |  | 5.12×10^-8^ |  |  |
| ***CDK12, NEUROD2*** rs11654954 |  |  |  | 2.18×10^-8^ |  |  |
| ***CDK12, NEUROD2*** rs11654954 |  |  |  | 3.75×10^-8^ |  |  |
| ***STARD3***  rs9972882 |  |  |  | 1.81×10^-8^ |  |  |
| **Kathiresan et al. (2008)** [[22](#_ENREF_22)] | | | | | | |
| *CELSR2, PSRC1, SORT1* rs646776 |  |  | 8×10^–8^ |  | European  (Denmark, Norway, Sweden)  n = 2,758  Type 2 diabetic cases and controls | 61.5 ± 10.5 |
| Unknown  rs599839 |  |  | 9×10^-8^ |  |  |  |
| *CILP2, PBX4* rs16996148 |  |  | 0.04 |  |  |  |
| *APOB*  rs693 |  |  | 7×10^-7^ |  |  |  |
| *APOE-C1-C4- C2* rs4420638 |  |  | 3×10^-13^ |  |  |  |
| *HMGCR*  rs12654264 |  |  | 0.0004 |  |  |  |
| *LDLR*  rs6511720 |  |  | 9×10^-7^ |  |  |  |
| *GALNT2*  rs4846914 |  | 3×10^-4^ |  |  |  |  |
| *ABCA1*  rs3890182 |  | 3×10^-5^ |  |  |  |  |
| *APOA1-C3-A4-A5, ZNF259, BUD13* rs28927680 |  | 0.31 |  |  |  |  |
| *CETP*  rs1800775 |  | 3×10^-13^ |  |  |  |  |
| *LIPC*  rs1800588 |  | 3×10^-5^ |  |  |  |  |
| *LIPG, ACAA2* rs2156552 |  | 0.02 |  |  |  |  |
| *LPL*  rs328 |  | 3×10^-4^ |  |  |  |  |
| *BCL7B, TBL2, MLXIPL* rs17145738 |  |  |  | 0.003 |  |  |
| *TRIB1*  rs17321515 |  |  |  | 7×10^-4^ |  |  |
| *GALNT2*  rs4846914 |  |  |  | 9×10^-5^ |  |  |
| *CILP2, PBX4* rs16996148 |  |  |  | 0.05 |  |  |
| *ANGPTL3, DOCK7, ATG4C*  rs12130333 |  |  |  | 0.0006 |  |  |
| *APOA1-C3-*  *A4-A5,*  *ZNF259,*  *BUD13*  rs28927680 |  |  |  | 6x10^-5^ |  |  |
| *APOB*  rs693 |  |  |  | 7×10^-4^ |  |  |
| *GCKR*  rs780094 |  |  |  | 4×10^-8^ |  |  |
| *LPL*  rs328 |  |  |  | 4×10^-7^ |  |  |
| **Oh et al. (2020)** [[23](#_ENREF_23)] | | | | | | |
| *GCKR*  rs780092 |  |  |  | 4.82×10^-9^ | Korean  Metabolic Syndrome cases and controls  Cases: n = 1,362  Controls: n = 6,061 | 53.5 ± 9.5 (Cases)  49.9 ± 10.2 (Controls) |
| *GCKR*  rs780093 |  |  |  | 2.55×10^-12^ |  |  |
| *GCKR*  rs780094 |  |  |  | 6.49×10^-12^ |  |  |
| *GCKR*  rs1260326 |  |  |  | 3.89×10^-12^ |  |  |
| *GCKR*  rs1260333 |  |  |  | 5.20×10^-12^ |  |  |
| *C2orf16*  rs1919127 |  |  |  | 1.18×10^-8^ |  |  |
| *C2orf16*  rs1919128 |  |  |  | 7.39×10^-9^ |  |  |
| *APOA5*  rs662799 |  |  |  | 4.97×10^-34^ |  |  |
| *APOA5*  rs2075291 |  |  |  | 3.67×10^-19^ |  |  |
| *APOA5*  rs2266788 |  |  |  | 9.26×10^-15^ |  |  |
| *ZPR1*  rs603446 |  |  |  | 6.24×10^-9^ |  |  |
| *ZPR1*  rs964184 |  |  |  | 1.47×10^-14^ |  |  |
| *BUD13*  rs2075295 |  |  |  | 4.56×10^-10^ |  |  |
| *BUD13*  rs11216126 |  |  |  | 1.34×10^-11^ |  |  |
| *BUD13*  rs1558861 |  |  |  | 5.85×10^-14^ |  |  |
| *APOA5*  rs662799 |  | 2.26×10^-16^ |  |  |  |  |
| *APOA5*  rs2075291 |  | 9.28×10^-9^ |  |  |  |  |
| *ALDH1A2*  rs4775041 |  | 2.28×10^-8^ |  |  |  |  |
| *ALDH1A2*  rs10468017 |  | 7.27×10^-8^ |  |  |  |  |
| *ALDH1A2*  rs1800588 |  | 5.62×10^-8^ |  |  |  |  |
| *HERPUD1*  rs72786786 |  | 1.65×10^-10^ |  |  |  |  |
| *HERPUD1*  rs173539 |  | 1.13×10^-8^ |  |  |  |  |
| *HERPUD1*  rs247616 |  | 1.29×10^-16^ |  |  |  |  |
| *HERPUD1*  rs247617 |  | 7.70×10^-17^ |  |  |  |  |
| *HERPUD1*  rs3764261 |  | 5.27×10^-17^ |  |  |  |  |
| *HERPUD1*  rs4783961 |  | 9.93×10^-9^ |  |  |  |  |
| *CETP*  rs708272 |  | 6.09×10^-8^ |  |  |  |  |
| *CETP*  rs7499892 |  | 1.57×10^-8^ |  |  |  |  |
| *CETP*  rs2303790 |  | 5.31×10^-11^ |  |  |  |  |
| **Zhu et al. (2017)** [[77](#_ENREF_77)] | | | | | | |
| *APOA5*  rs651821 |  |  |  | 7.8×10^-16^ | Han Chinese  Metabolic Syndrome cases and controls  Cases: n = 862  Controls: n = 880 | 59.9 ± 10.7 (Cases)  55.3 ± 12.0 (Controls) |
| *BUD13*  rs180326 |  |  |  | 3.9×10^-7^ |  |  |
| *TPBG\|\|UBE2CBP* rs209411 |  | 9.5×10^-6^ |  |  |  |  |
| *TLE1\|\|FLJ43950* rs7864030 |  | 1.0×10^-6^ |  |  |  |  |
| *APOA5*  rs651821 |  | 6.5×10^-7^ |  |  |  |  |
| *STRBP\|\|CRB2*  rs10985976 |  |  | 6.2×10^-6^ |  |  |  |
| *MYO19*  rs12602787 |  |  | 3.6×10^-6^ |  |  |  |
| *CUX1*  rs420437 |  |  | 3.3×10^-6^ |  |  |  |
| *APOC1*  rs445925 |  |  | 4.1×10^-12^ |  |  |  |
| *APOA5*  rs651821 |  |  |  | 7.8×10^-16^ |  |  |
| **Wu et al. (2013)** [[24](#_ENREF_24)] | | | | | | |
| ***APOE***  rs7412 |  |  | 2.7×10^-53^ |  | Filipino Women  Mothers: n = 1,782  Offspring: n = 1,719 | 48.4 ± 6.1 (Mothers)  21.5 ± 0.3  (Offspring) |
| ***APOE***  rs7412 | 1.5×10^-30^ |  |  |  |  |  |
| ***APOA5***  rs662799 |  |  |  | 5.7×10^-24^ |  |  |
| ***GCKR***  rs780092 |  |  |  | 1.8×10^-09^ |  |  |
| ***CETP***  rs1800775 |  | 3.4×10^-9^ |  |  |  |  |
| ***TOM1***  rs138777 |  | 4.0×10^-5^ |  |  |  |  |
| ***LPL***  rs328 |  |  |  | 1.6×10^-3^ |  |  |
| ***MLXIPL***  rs17145738 |  |  |  | 4.2×10^-3^ |  |  |
| ***ANGPTL3***  rs2131925 |  |  |  | 5.1×10^-3^ |  |  |
| ***LIPC***  rs588136 |  | 1.5×10^-12^ |  |  |  |  |
| ***LIPG***  rs2156552 |  | 4.6×10^-3^ |  |  |  |  |
| ***MMAB-MVK*** rs10774708 |  | 0.011 |  |  |  |  |
| ***ABO***  rs2519093 |  |  | 3.0×10^-5^ |  |  |  |
| ***APOB***  rs1367117 |  |  | 7.4×10^-3^ |  |  |  |
| ***TIMD4***  rs6882076 | 4.0×10^-3^ |  |  |  |  |  |
| ***DNAH11***  rs5008148 | 0.012 |  |  |  |  |  |
| **Chasman et al. (2009)** [[92](#_ENREF_92)] | | | | | | |
| *PCSK9*  rs11591147 |  |  | 1.6×10^-7^ |  | White American Women: n=6,382 | 52 (49–58) |
| *CELSR2, PSRC1, SORT1*  rs646776 |  |  | 4.9×10^-19^ |  |  |  |
| *APOB*  rs506585 |  |  | 9.3×10^-09^ |  |  |  |
| *GCKR*  rs1260326 |  |  |  | 1.3×10^-16^ |  |  |
| *LPL*  rs328 |  |  |  | 4.7×10^-11^ |  |  |
| *LPL*  rs331 |  | 9.1×10^-7^ |  | 1.7×10^-9^ |  |  |
| *APOA5-APOA1* rs3135506 |  |  |  | 5.5×10^-12^ |  |  |
| *APOA5-APOA1* rs662799 |  |  |  | 2.9×10^-15^ |  |  |
| *APOA5-APOA1* rs12225230 |  | 5.3×10^-5^ |  |  |  |  |
| *LIPC*  rs1532085 |  | 1.3×10^-10^ |  |  |  |  |
| **Keller et al.** **(2013)** [[93](#_ENREF_93)] | | | | | | |
| *TMPRSS2*  rs2298857 |  | 3.85×10^-7^ |  |  | Eastern Germany  (The Sorbs)  n = 839 | 46 ± 16 |
| *SSTR1*  rs1954021 |  | 7.39×10^-6^ |  |  |  |  |
| *FRMD1*  rs3816859 |  | 8.79×10^-6^ |  |  |  |  |
| *SOX6*  rs297360 |  | 9.02×10^-6^ |  |  |  |  |
| Unknown  rs7081043 |  | 9.68×10^-6^ |  |  |  |  |
| *ABHB5*  rs883212 |  |  | 4.80×10^-7^ |  |  |  |
| *MIST*  rs10488946 |  |  | 1.78×10^-6^ |  |  |  |
| *HTR5A*  rs1730206 |  |  | 2.91×10^-6^ |  |  |  |
| *FAM112A*  rs3127065 |  |  | 3.03×10^-6^ |  |  |  |
| *ABHB5*  rs17583742 |  |  | 3.08×10^-6^ |  |  |  |
| *TNFSF4*  rs10127728 |  |  | 6.41×10^-6^ |  |  |  |
| *PCSK5*  rs10869740 |  |  |  | 1.86×10^-6^ |  |  |
| *REPS1*  rs9484217 |  |  |  | 4.66×10^-6^ |  |  |
| *C6ORF166*  rs4707385 |  |  |  | 4.93×10^-6^ |  |  |
| **Waterworth et al. (2010)^c^** [[25](#_ENREF_25)] | | | | | | |
| ***PCSK9***  rs11206510 |  | 0.52 | 1.2×10^−10^ | 0.04 | White European  (British, Swedish, Finnish and Italian)  n = 17,543 | 31^d^ (0.0) – 75^d^ (5.0) |
| ***CELSR2***  rs660240 |  | 0.22 | 1.2×10^−26^ | 0.56 |  |  |
| ***APOB***  rs515135 |  | 0.47 | 2.4×10^−20^ | 0.25 |  |  |
| ***HMGCR***  rs12916 |  | 0.80 | 1.4×10^−11^ | 0.64 |  |  |
| ***TRIB1***  rs2954021 |  | 1.3×10^−4^ | 1.4×10^−7^ | 6.3×10^−11^ |  |  |
| ***BUD13, ZNF259, APOA5-A4-C3-A1*** rs1558861 |  | 1.7×10^−7^ | 2.0×10^−6^ | 2.0×10^−30^ |  |  |
| ***LDLR***  rs2738459 |  | 0.34 | 6.6×10^−6^ | 0.31 |  |  |
| ***SF4–CILP2*** rs10401969 |  | 0.26 | 9.5×10^−12^ | 8.4×10^−14^ |  |  |
| ***APOE-C1-C4-C2*** rs4420638 |  | 2.0×10^−7^ | 1.7×10^−40^ | 5.5×10^−7^ |  |  |
| ***GALNT2***  rs10489615 |  | 3.8×10^−9^ | 0.25 | 2.4×10^−4^ |  |  |
| ***APOB***  rs11902417 |  | 3.7×10^−7^ | 4.0×10^−3^ | 2.7×10^−7^ |  |  |
| ***LPL***  rs325 |  | 7.8×10^−25^ | 0.34 | 4.9×10^−24^ |  |  |
| ***ABCA1***  rs3890182 |  | 4.7 × 10^−7^ | 0.43 | 0.16 |  |  |
| ***ZNF259, APOA5-A4-C3-A1***  rs964184 |  | 1.6 × 10^−11^ | 6.4 × 10^−6^ | 9.0 × 10^−53^ |  |  |
| ***MYO1H, KCTD10, UBE3B, MMAB, MVK*** rs9943753 |  | 3.2×10^−6^ | 0.20 | 0.51 |  |  |
| ***LIPC***  rs261334 |  | 4.9×10^−22^ | 0.65 | 0.01 |  |  |
| ***CETP***  rs9989419 |  | 1.3×10^−32^ | 0.58 | 0.67 |  |  |
| ***GFOD2–LCAT*** rs12449157 |  | 2.3×10^−7^ | 0.31 | 0.02 |  |  |
| ***LIPG***  rs2156552 |  | 1.7×10^−12^ | 0.01 | 0.02 |  |  |
| ***DOCK7, ANGPTL3*** rs1168013 |  | 0.97 | 6.7 × 10^−3^ | 6.4×10^−8^ |  |  |
| ***APOB***  rs6544366 |  | 5.3×10^−7^ | 3.8 × 10^−3^ | 1.9×10^−7^ |  |  |
| ***GCKR***  rs1260333 |  | 0.08 | 0.36 | 1.7×10^−19^ |  |  |
| ***BAZ1B, BCL7B, TBL2, MLXIPL***  rs1178979 |  | 8.0 × 10^−3^ | 2.5 × 10^−3^ | 2.3×10^−12^ |  |  |
| ***LPL***  rs10105606 |  | 1.7×10^−14^ | 0.94 | 3.6×10^−25^ |  |  |
| ***TRIB1***  rs2954029 |  | 4.5×10^−5^ | 9.2×10^−7^ | 1.8×10^−11^ |  |  |
| ***BUD13, ZNF259, APOA5-A4-C3-A1*** rs4938303 |  | 9.6×10^−8^ | 0.02 | 4.1×10^−21^ |  |  |
| ***CETP, LOC100130044, NLRC5***  rs16965220 |  | 0.04 | 0.01 | 9.6×10^−6^ |  |  |
| ***CILP2–ZNF101*** rs2304130 |  | 0.55 | 1.1×10^−7^ | 3.9×10^−8^ |  |  |
| **Dumitrescu et al. (2011)** [[94](#_ENREF_94)] | | | | | | |
| *SGSM2*  rs2429917 |  |  | 7.01×10^-06^ |  | African  n = 66 | 7.2 ± 4 |
| Intergenic  rs12190789 |  |  | 3.32×10^-06^ |  |  |  |
| *CD96*  rs16858329 |  |  |  | 4.29×10^-06^ |  |  |
| Intergenic  rs6477578 |  |  |  | 6.13×10^-06^ | European  n = 282 | 7.0 ± 5 |
| *FRMD3*  rs10868008 |  |  | 1.66×10^-06^ |  | Mexican  n = 63 | 6.4 ± 4 |
| *FRMD3*  rs11140077 |  |  | 1.66×10^-06^ |  |  |  |
| **Hiura et al. (2009)** [[26](#_ENREF_26)] | | | | | | |
| *CETP*  rs3764261 |  | 6.17^e^ |  |  | Japanese  n = 900 | 59.8 ± 7.3 (Men)  58.2 ± 6.8 (Women) |
| Unknown  rs10945991 |  | 5.90^e^ |  |  |  |  |
| *ZNF665*  rs6509732 |  | 5.50^e^ |  |  |  |  |
| *SLC23A2*  rs6133175 |  | 4.98^e^ |  |  |  |  |
| *FLJ45139*  rs467571 |  | 4.97^e^ |  |  |  |  |
| Unknown  rs10485472 |  | 4.90^e^ |  |  |  |  |
| Unknown  rs1469918 |  | 4.82^e^ |  |  |  |  |
| Unknown  rs6790597 |  | 4.78^e^ |  |  |  |  |
| Unknown  rs12225506 |  | 4.72^e^ |  |  |  |  |
| *BCL2L14*  rs1544669 |  | 4.70^e^ |  |  |  |  |
| Unknown  rs12206635 |  | 4.64^e^ |  |  |  |  |
| *C14orf118*  rs2246454 |  | 4.62^e^ |  |  |  |  |
| Unknown  rs980861 |  | 4.58^e^ |  |  |  |  |
| *RAP1GAP*  rs12134357 |  | 4.49^e^ |  |  |  |  |
| *TMEM200A* rs17059002 |  | 4.45^e^ |  |  |  |  |
| *PSMB6, PLD2* rs11654690 |  | 4.44^e^ |  |  |  |  |
| *CCT8L2*  rs2236639 |  | 4.42^e^ |  |  |  |  |
| *ACCN1*  rs280049 |  | 4.34^e^ |  |  |  |  |
| *ESRRG*  rs7547186 |  | 4.31^e^ |  |  |  |  |
| Unknown  rs7550051 |  | 4.31^e^ |  |  |  |  |
| Unknown  rs4656747 |  | 4.30^e^ |  |  |  |  |
| *ADARB2*  rs2813397 |  | 4.29^e^ |  |  |  |  |
| Unknown  rs12586473 |  | 4.27^e^ |  |  |  |  |
| *SLC23A2*  rs3914810 |  | 4.27^e^ |  |  |  |  |
| Unknown  rs10493889 |  | 4.24^e^ |  |  |  |  |
| Unknown  rs9956878 |  | 4.17^e^ |  |  |  |  |
| *CLASP1*  rs10496565 |  | 4.14^e^ |  |  |  |  |
| *TMC2*  rs4815298 |  | 4.13^e^ |  |  |  |  |
| Unknown  rs6990139 |  | 4.13^e^ |  |  |  |  |
| *GABRR1*  rs9359845 |  | 4.12^e^ |  |  |  |  |
| *UGT3A1*  rs2242225 |  | 4.11^e^ |  |  |  |  |
| *TAF1B*  rs450286 |  | 4.04^e^ |  |  |  |  |
| Unknown  rs12453139 |  | 4.04^e^ |  |  |  |  |
| Unknown  rs4404877 |  | 4.01^e^ |  |  |  |  |
| **Smith et al. (2010)** [[27](#_ENREF_27)] | | | | | | |
| *HERPUD1/CETP* rs247616 |  | 6.6×10^-7^ |  |  | Individuals of European ancestry in the USA  n = 525 | 4 – 48 |
| *MRPS6/KCNE2* rs8131349 |  |  | 1.4×10^-8^ |  |  |  |
| *APOE/APOC1*  rs7412 |  |  | 1.6×10^-8^ |  |  |  |
| *C6orf170/GJA1* rs7738656 |  |  | 2.5×10^-7^ |  |  |  |
| *KIF4B/SGCD* rs10044666 |  |  | 4.7×10^-7^ |  |  |  |
| *MRPS6/KCNE2* rs8131349 | 4.6×10^-8^ |  |  |  |  |  |
| *ABLIM2*  rs6829649 | 9.6×10^-8^ |  |  |  |  |  |
| *C6orf170/GJA1* rs7738656 | 2.1×10^-7^ |  |  |  |  |  |
| *ST3GAL1/ZFAT* rs4897695 | 2.7×10^-7^ |  |  |  |  |  |
| **Tan et al. (2012)** [[40](#_ENREF_40)] | | | | | | |
| *RYR2*  rs16835705 | 1.15×10^-6^ |  |  |  | Chinese Men  n = 1,999 | 37.54 ± 11.10 |
| *LPL*  rs328 | 4.90×10^-8^ |  |  |  |  |  |
| *SLCO5A1*  rs10504457 | 1.05×10^-6^ |  |  |  |  |  |
| *APOA5*  rs651821 | 6.10×10^-15^ |  |  |  |  |  |
| *ALDH2*  rs671 | 4.85×10^-6^ |  |  |  |  |  |
| Intergenic  rs1532085 | 1.01×10^-6^ |  |  |  |  |  |
| *TOMM40*  rs157581 | 8.48×10^-7^ |  |  |  |  |  |
| **Zabaneh et al. (2010)** [[28](#_ENREF_28)] | | | | | | |
| *CETP*  rs3764261 |  | 1.3×10^-48^ |  |  | Men of Indian Asian descent from West London  n = 2,684 | 50.0 ± 11.0 |
| *CETP*  rs9989419 |  | 1.4×10^-20^ |  |  |  |  |
| *LPL*  rs2083637 |  | 1.9×10^-10^ |  |  |  |  |
| *LPL*  rs4523270 |  | 1.0×10^-07^ |  |  |  |  |
| *FLJ41733*  rs496300 |  | 3.9×10^-07^ |  |  |  |  |
| *FADS1*  rs174546 |  | 6.0×10^-07^ |  |  |  |  |
| *FADS2*  rs1535 |  | 6.5×10^-07^ |  |  |  |  |
| **Lettre et al. (2011)**^c^ [[29](#_ENREF_29)] | | | | | | |
| *GALNT2*  rs2144300 |  | 0.0015 |  |  | African American  n = 8,090 | 24.4^d^ ± 3.8 –  73.4^d^ ± 2.9 |
| *PPP1R3B*  rs9987289 |  | 4.3×10^-5^ |  |  |  |  |
| *LPL*  rs10503669 |  | 7.2×10^-5^ |  |  |  |  |
| Unknown  rs10096633 |  | 1.5×10^-9^ |  |  |  |  |
| *ABCA1*  rs3905000 |  | 0.054 |  |  |  |  |
| Unknown  rs13284054 |  | 0.0011 |  |  |  |  |
| *FADS1, FADS2, FADS3*  rs174547 |  | 0.068 |  |  |  |  |
| Unknown  rs1535 |  | 6.7×10^-5^ |  |  |  |  |
| *LIPC*  rs1800588 |  | 1.5×10^-8^ |  |  |  |  |
| Unknown  rs8034802 |  | 1.3×10^-9^ |  |  |  |  |
| *CETP*  rs3764261 |  | 8.6×10^-18^ |  |  |  |  |
| Unknown  rs247617 |  | 1.2×10^-43^ |  |  |  |  |
| *LCAT*  rs255052 |  | 6.6×10^-11^ |  |  |  |  |
| *PLTP*  rs7679 |  | 0.22 |  |  |  |  |
| Unknown  rs6065904 |  | 7.4×10^-5^ |  |  |  |  |
| *DOCK7*  rs10889353 |  |  | 0.0040 |  |  |  |
| Unknown  rs10889335 |  |  | 1.2×10^-4^ |  |  |  |
| *CELSR2, PSRC1, SORT1*  rs12740374 |  |  | 1.3×10^-16^ |  |  |  |
| *PCSK9*  rs10493178 |  |  | 4.7×10^-12^ |  |  |  |
| *APOB*  rs562338 |  |  | 3.1×10^-7^ |  |  |  |
| Unknown  rs503662 |  |  | 2.5×10^-9^ |  |  |  |
| *LDLR*  rs6511720 |  |  | 7.2×10^-8^ |  |  |  |
| *APOE, APOC1, APOC4, APOC2* rs1160985 |  |  | 7.2×10^-21^ |  |  |  |
| **Carlson et al. (2020)** [[30](#_ENREF_30)] | | | | | | |
| *APOB*  rs754523 | 6.25×10^-6^ |  |  |  | Samoans  n =2,849 | 44.8 ± 11.1 (Women)  45.6 ± 11.1 (Men) |
| *PDE4D*  rs7711093 | 3.01×10^-6^ |  |  |  |  |  |
| *LUCAT1*  rs10072084 | 9.48×10^-6^ |  |  |  |  |  |
| *FILIP1*  rs2951921 | 9.04×10^-7^ |  |  |  |  |  |
| *ZHX2*  rs7841763 | 4.82×10^-6^ |  |  |  |  |  |
| *APOA1*  rs964184 | 5.37×10^-5^ |  |  |  |  |  |
| *SIRT2*  rs10405150 | 6.34×10^-6^ |  |  |  |  |  |
| *ZNF283*  rs16976816 | 9.78×10^-6^ |  |  |  |  |  |
| *APOE*  rs1160985 | 2.13×10^-13^ |  |  |  |  |  |
| *STON1-GTF2A1L* rs6739536 |  | 1.58×10^-6^ |  |  |  |  |
| *MGAT1*  rs1038143 |  | 3.72×10^-6^ |  |  |  |  |
| *AKAP7*  rs3777486 |  | 3.09×10^-6^ |  |  |  |  |
| *CSMD1*  rs1626142 |  | 7.67×10^-6^ |  |  |  |  |
| *RAB21*  rs328733 |  | 2.57×10^-6^ |  |  |  |  |
| *ZNF10*  rs2292029 |  | 4.05×10^-6^ |  |  |  |  |
| *HS6ST3*  rs16953620 |  | 8.48×10^-6^ |  |  |  |  |
| *LIPC*  rs10438284 |  | 4.00×10^-7^ |  |  |  |  |
| *CETP*  rs289708 |  | 1.19×10^-11^ |  |  |  |  |
| *LIPG*  rs16950739 |  | 1.07×10^-7^ |  |  |  |  |
| *APOE*  rs1160985 |  | 0.003 |  |  |  |  |
| *CDH4*  rs817687 |  | 2.31×10^-6^ |  |  |  |  |
| *APOB*  rs754523 |  |  | 3.25×10^-6^ |  |  |  |
| *KALRN*  rs6789134 |  |  | 3.22×10^-6^ |  |  |  |
| *ZHX2*  rs7841763 |  |  | 1.80×10^-6^ |  |  |  |
| *SH2D4B*  rs10509415 |  |  | 7.96×10^-6^ |  |  |  |
| *ALG10*  rs3912355 |  |  | 2.12×10^-6^ |  |  |  |
| *ALG10B*  rs10880642 |  |  | 5.56×10^-6^ |  |  |  |
| *CPNE8*  rs11169807 |  |  | 4.77×10^-6^ |  |  |  |
| *LINC02408*  rs17104016 |  |  | 9.29×10^-6^ |  |  |  |
| *LINC00922*  rs254371 | 9.04×10^-6^ |  |  |  |  |  |
| *ZNF283*  rs16976816 | 1.78×10^-6^ |  |  |  |  |  |
| *APOE*  rs1160985 | 2.61×10^-20^ |  |  |  |  |  |
| *GCKR*  rs780094 |  |  |  | 9.84×10^-7^ |  |  |
| *CD200*  rs2399416 |  |  |  | 5.12×10^-6^ |  |  |
| *SPIN1*  rs7861888 |  |  |  | 4.24×10^-6^ |  |  |
| *APOA1*  rs964184 |  |  |  | 2.37×10^-17^ |  |  |
| *KIRREL3*  rs3018434 |  |  |  | 4.16×10^-6^ |  |  |
| *APOE*  rs1160985a |  |  |  | 0.312 |  |  |
| **Kurano et al. (2016)** [[31](#_ENREF_31)] | | | | | | |
| *CELSR2*  rs660240 |  |  | 4.56×10^-5^ |  | Japanese, healthy individuals  n = 2,994 | 20 – Over 40 |
| *CELSR2*  rs646776 |  |  | 4.93×10^-5^ |  |  |  |
| *CELSR2*  rs629301 |  |  | 3.22×10^-5^ |  |  |  |
| *PSRC1*  rs599839 |  |  | 7.86×10^-5^ |  |  |  |
| *CELSR2*  rs629301 |  |  | 3.22×10^-5^ |  |  |  |
| *APOB*  rs1367117 |  |  | 3.19×10^-2^ |  |  |  |
| *HMGCR*  rs12916 |  |  | 1.19×10^-4^ |  |  |  |
| *HMGCR*  rs3846662 |  |  | 1.10×10^-4^ |  |  |  |
| *HMGCR*  rs12916 |  |  | 1.19×10^-4^ |  |  |  |
| *TIMD4-HAVCR1* rs58198139 |  |  | 2.25×10^-4^ |  |  |  |
| *TIMD4*  rs6882076 |  |  | 2.86×10^-4^ |  |  |  |
| *HPR*  rs2000999 |  |  | 9.65×10^-3^ |  |  |  |
| *TOMM40*  rs1160985 |  |  | 2.69×10^-6^ |  |  |  |
| *APOC1*  rs4420638 |  |  | 1.39×10^-5^ |  |  |  |
| *PABPC4*  rs4660293 |  | 4.24×10^-2^ |  |  |  |  |
| *LOC100130996* rs1779824 |  | 2.45×10^-2^ |  |  |  |  |
| *GALNT2*  rs2144300 |  | 1.69×10^-2^ |  |  |  |  |
| *LPL*  rs327 |  | 2.58×10^-4^ |  |  |  |  |
| *LPL*  rs328 |  | 6.47×10^-7^ |  |  |  |  |
| *LPL-RPL30P9* rs17482753 |  | 1.33×10^-6^ |  |  |  |  |
| *LPL*  rs328 |  | 6.47×10^-7^ |  |  |  |  |
| *ABCA1*  rs12686004 |  | 3.05×10^-2^ |  |  |  |  |
| *ABCA1*  rs1883025 |  | 5.33×10^-3^ |  |  |  |  |
| *FADS1*  rs174548 |  | 6.81×10^-3^ |  |  |  |  |
| *FADS1*  rs174547 |  | 6.86×10^-3^ |  |  |  |  |
| *FADS1*  rs174546 |  | 9.05×10^-3^ |  |  |  |  |
| *MMAB*  rs7134594 |  | 1.38×10^-2^ |  |  |  |  |
| *NCOR2-SCARB1* rs838880 |  | 1.10×10^-3^ |  |  |  |  |
| *LIPC*  rs1800588 |  | 2.14×10^-4^ |  |  |  |  |
| *RPL28P4-LIPC* rs1532085 |  | 7.30×10^-4^ |  |  |  |  |
| *LIPC*  rs1800588 |  | 2.14×10^-4^ |  |  |  |  |
| *CETP*  rs12708980 |  | 4.79×10^-3^ |  |  |  |  |
| *HERPUD1-CETP* rs9989419 |  | 1.12×10^-6^ |  |  |  |  |
| *HERPUD1-CETP* rs3764261 |  | 1.18×10^-16^ |  |  |  |  |
| *CETP*  rs1800775 |  | 1.01×10^-2^ |  |  |  |  |
| *NUTF2*  rs2271293 |  | 4.76×10^-2^ |  |  |  |  |
| *PSKH1*  rs16942887 |  | 4.98×10^-2^ |  |  |  |  |
| *HNF4A*  rs1800961 |  | 6.83×10^-3^ |  |  |  |  |
| *DOCK7*  rs1748195 |  |  |  | 0.001273 |  |  |
| *GCKR*  rs780092 |  |  |  | 0.01145 |  |  |
| *GCKR*  rs1260326 |  |  |  | 0.00072 |  |  |
| *GCKR*  rs780094 |  |  |  | 0.003526 |  |  |
| *TIMD4*  rs6882076 |  |  |  | 0.002649 |  |  |
| *TBL2*  rs17145738 |  |  |  | 0.000647 |  |  |
| *TBL2*  rs2286276 |  |  |  | 0.000277 |  |  |
| *LPL*  rs327 |  |  |  | 2.81×10^-8^ |  |  |
| *LPL-RPL30P9* rs17410996 |  |  |  | 1.75×10^-8^ |  |  |
| *LPL-RPL30P9* rs17410996 |  |  |  | 1.75×10^-8^ |  |  |
| *LPL-RPL30P9* rs10105606 |  |  |  | 2.24×10^-7^ |  |  |
| *LPL-RPL30P9* rs7841189 |  |  |  | 1.75×10^-8^ |  |  |
| *LPL-RPL30P9* rs17482753 |  |  |  | 2.26×10^-8^ |  |  |
| *LPL*  rs328 |  |  |  | 4.47×10^-8^ |  |  |
| *XKR6*  rs7819412 |  |  |  | 0.01766 |  |  |
| *TRIB1-LINC00861* rs17321515 |  |  |  | 0.0137 |  |  |
| *SIK3*  rs2075292 |  |  |  | 8.80×10^-7^ |  |  |
| *APOA5*  rs651821 |  |  |  | 1.01×10^-30^ |  |  |
| *RPL15P15, BUD13* rs4938303 |  |  |  | 0.01347 |  |  |
| **Coram et al (2013)** [[32](#_ENREF_32)] | | | | | | |
| *PCSK9*  rs17111684 |  |  | 2.40×10^-17^ |  | African American  Women  HDL: n = 7,917  LDL: n = 7,861  TG: n= 7,918 | 50–79 |
| *APOB*  rs12713956 |  |  | 3.74×10^-08^ |  |  |  |
| *GCKR*  rs4665972c |  |  |  | 1.05×10^-08^ |  |  |
| *ABCG8*  rs4245791 |  |  | 1.24×10^-09^ |  |  |  |
| *CD36*  rs2366858 |  | 5.59×10^-10^ |  |  |  |  |
| *PPP1R3B*  rs1461729 |  | 7.39×10^-09^ |  |  |  |  |
| *LPL*  rs326 |  | 1.23×10^-08^ |  |  |  |  |
| *LPL*  rs326 |  |  |  | 1.02×10^-08^ |  |  |
| *APOA/APOC* rs6589566 |  |  |  | 4.99×10^-14^ |  |  |
| *APOA/APOC*  chr11: 116,799,496 |  | 1.08×10^-12^ |  |  |  |  |
| *CETP*  rs247617 |  | 1.48×10^-44^ |  |  |  |  |
| *LDLR*  rs17249141 |  |  | 2.43×10^-17^ |  |  |  |
| *LOC55908*  rs12979813 |  | 1.99×10^-09^ |  |  |  |  |
| *APOE*  rs1160985 |  |  | 1.87×10^-21^ |  |  |  |
| *APOC1*  rs12721054 |  |  |  | 2.86×10^-19^ |  |  |
| *PPP1R3B*  rs1461729 |  | 7.39×10^-09^ |  |  |  |  |
| Unknown  rs13046373 |  | 2.26×10^-08^ |  |  |  |  |
| *GCKR*  rs780094 |  |  |  | 7.35×10^-09^ | Hispanic American  Women  n = 3,506 (HDL)  n = 3,425 (LDL)  n= 3,506 (TG | 50–79 |
| *LPL*  rs17410962 |  |  |  | 7.35×10^-09^ |  |  |
| *APOA/APOC*  rs964184 |  | 2.81×10^-12^ |  |  |  |  |
| *APOA/APOC*  rs964184 |  |  |  | 3.66×10^-33^ |  |  |
| *CETP*  rs247617 |  | 3.48×10^-16^ |  |  |  |  |
| **Aulchenko et al. (2008)**^c^ [[33](#_ENREF_33)] | | | | | | |
| *TMEM57*  rs10903129 | 5.4×10^-10^ | 0.02 | 1.8×10^-5^ | 0.48 | Different European countries including Austria and Finland  n= 17,797–22,562 | 18–104 |
| *DOCK7*  rs1167998 | 6.4×10^-10^ | 3.8×10^-3^ | 1.1×10^-5^ | 2.0×10^-12^ |  |  |
| *DOCK7*  rs10889353 | 3.7×10^-12^ | 1.8×10^-3^ | 7.9×10^-6^ | 8.2×10^-11^ |  |  |
| *CELSR2*  rs646776 | 8.5×10^-22^ | 6.2×10-^3^ | 7.8×10^-23^ | 0.63 |  |  |
| *APOB*  rs693 | 8.7×10^-23^ | 1.3×10^-7^ | 3.6×10^-17^ | 1.4×10^-4^ |  |  |
| *APOB*  rs6754295 | 1.8×10^-6^ | 4.4×10^-8^ | 1.6×10^-7^ | 2.5×10^-8^ |  |  |
| *APOB*  rs673548 | 7.4×10^-05^ | 7.4×10^-7^ | 3.6×10^-5^ | 1.1×10^-8^ |  |  |
| *GCKR*  rs780094 | 0.02 | 0.12 | 0.85 | 3.1×10^-20^ |  |  |
| *ABCG5*  rs6756629 | 1.5×10^-11^ | 0.74 | 2.6×10^-10^ | 0.26 |  |  |
| *HMGCR*  rs3846662 | 2.5×10^-19^ | 0.57 | 1.5×10^-11^ | 0.03 |  |  |
| *DNAH11*  rs12670798 | 9.2×10^-7^ | 0.14 | 6.1×10^-9^ | 0.68 |  |  |
| *MLXIPL*  rs2240466 | 0.80 | 0.02 | 0.44 | 1.1×10^-12^ |  |  |
| *LPL*  rs2083637 | 0.73 | 5.5×10^-18^ | 0.76 | 1.0×10^-14^ |  |  |
| *LPL*  rs10096633 | 0.91 | 6.1×10^-16^ | 0.53 | 1.9×10^-18^ |  |  |
| *TRIB1*  rs6987702 | 3.3×10^-9^ | 0.44 | 2.9×10^-6^ | 5.2×10^-5^ |  |  |
| *ABCA1*  rs3905000 | 5.0×10^-5^ | 8.6×10^-13^ | 0.90 | 0.20 |  |  |
| *MADD-FOLH1* rs7395662 | 0.63 | 6.0×10^-11^ | 0.31 | 0.54 |  |  |
| *FADS2/3*  rs174570 | 1.5×10^-10^ | 3.9×10^-6^ | 4.4×10^-13^ | 2.9×10^-5^ |  |  |
| *APO(A1/A4/A5/C3)* rs12272004 | 7.3×10^-7^ | 0.01 | 9.9×10^-4^ | 5.4×10^-13^ |  |  |
| *LIPC*  rs1532085 | 3.7×10^-7^ | 9.7×10^-36^ | 0.60 | 0.33 |  |  |
| *CETP*  rs1532624 | 0.01 | 9.4×10^-94^ | 3.3×10^-3^ | 1.1×10^-3^ |  |  |
| *CTCF-PRMT8* rs2271293 | 0.14 | 8.3×10^-16^ | 0.33 | 0.04 |  |  |
| *LIPG*  rs4939883 | 2.4×10^-7^ | 1.6×10^-11^ | 0.06 | 0.11 |  |  |
| *LDLR*  rs2228671 | 9.3×10^-24^ | 0.54 | 4.2×10^-14^ | 0.59 |  |  |
| *NCAN*  rs2304130 | 2.0×10^-15^ | 0.75 | 1.5×10^-7^ | 2.9×10^-6^ |  |  |
| *TOMM40-APOE* rs2075650 | 2.9×10^-19^ | 1.9×10^-4^ | 9.3×10^-19^ | 2.4×10^-4^ |  |  |
| *TOMM40-APOE* rs157580 | 5.1×10^-17^ | 3.6×10^-7^ | 2.1×10^-19^ | 1.2×10^-8^ |  |  |
| *TOMM40-APOE*  rs439401 | 3.7×10^-4^ | 2.7×10^-3^ | 1.1×10^-2^ | 1.8×10^-9^ |  |  |
| **Adeyemo et al (2012)** [[34](#_ENREF_34)] | | | | | | |
| *CILP2/SF4*  rs10401969 |  |  |  | 0.006 | African Americans non-diabetic  n = 887 | 45.5 ± 12.4 (Men)  46.4 ± 13.3 (Women) |
| *STARD3*  rs11869286 |  | 0.02 |  |  |  |  |
| *LPL*  rs12678919 |  |  |  | 0.01 |  |  |
| *CYP7A1*  rs2081687 |  |  | 0.04 |  |  |  |
| *ANGPTL3*  rs2131925 | 0.04 |  |  |  |  |  |
| *APOE*  rs4420638 |  | 0.02 |  |  |  |  |
| *SORT1*  rs629301 |  |  | 0.005 |  |  |  |
| *IRS1*  rs2943652 |  | 0.01 |  |  |  |  |
| *CETP*  rs173539 |  | 0.0003 |  |  |  |  |
| *CETP*  rs1800775 |  | 0.03 |  |  |  |  |
| *CETP*  rs4783961 |  | 0.0009 |  |  |  |  |
| *UBASH3B*  rs6589939 | 0.005 |  |  |  |  |  |
| **Hoffmann et al (2018)** [[95](#_ENREF_95)] | | | | | | |
| *FAF1*  rs144432213 |  |  |  | 6.1×10^-9^ | Non-Hispanic white  Latino  East Asian  African American  South Asian  n = 94, 674 | 45.2 – 57.8 |
| *NFIA*  rs55878063 |  | 1.5×10^-8^ |  |  |  |  |
| *DR1*  rs145882729 |  | 6.1×10^-10^ |  |  |  |  |
| *PIGC*  rs7519429 |  |  |  | 1.4×10^-10^ |  |  |
| *LAMC1*  rs4651135 | 1.2×10^-8^ |  | h |  |  |  |
| *OPTC*  rs6695980 |  |  |  | 2.8×10^-8^ |  |  |
| *RP11-95P13.1* rs2791547 |  |  |  | 3.1×10^-10^ |  |  |
| *TIA1*  rs2706770 | 1.1×10^-8^ |  |  |  |  |  |
| *TGOLN2*  rs10198423 |  | 5.1×10^-9^ |  |  |  |  |
| *AC096670.3* rs77004761 |  |  |  | 6.7 ×10^-10^ |  |  |
| *TIA1*  rs2706770 | 1.1×10^-8^ |  |  |  |  |  |
| *TGOLN2*  rs10198423 |  | 5.1×10^-9^ |  |  |  |  |
| *AC096670.3* rs77004761 |  |  |  | 6.7 ×10^-10^ |  |  |
| *IL1RN*  rs55709272 | 1.8 × 10^-8^ |  |  |  |  |  |
| *KPNA1*  rs72285796 |  |  | 4.3 ×10^-12^ |  |  |  |
| *RP11-550I24.2* rs78086267 |  |  |  | 1.8×10^-8^ |  |  |
| *ETV5*  rs112545201 |  | 2.5×10^-19^ |  |  |  |  |
| *TMPRSS11E* rs13114070 | 1.6×10^-8^ |  |  |  |  |  |
| *TET2*  rs201330646 |  | 2.2×10^-9^ |  |  |  |  |
| *FCHO2*  rs62362194 |  | 4.2×10^-8^ |  |  |  |  |
| *C5orf56*  rs2522061 |  |  | 4.4×10^-11^ |  |  |  |
| *RP11-32D16.1* rs1651274 |  | 3×10^-9^ |  |  |  |  |
| *GNMT*  rs10948059 |  | 8.1×10^-9^ |  |  |  |  |
| *GSTA5*  rs12529923 |  | 1.4×10^-8^ |  |  |  |  |
| *RP3-332B22.1* rs181937009 |  |  |  | 1.7×10^-9^ |  |  |
| *RGS17*  rs1281955 |  | 2.2×10^-8^ |  |  |  |  |
| *TRGC2*  rs2534596 |  | 2.1×10^-8^ |  |  |  |  |
| *CALCR*  rs2299247 |  |  |  | 1.3×10^-9^ |  |  |
| *SH2D4A*  rs2958557 |  |  |  | 2.6×10^-8^ |  |  |
| *SNTB1*  rs13248499 |  | 1.4×10^-11^ |  |  |  |  |
| *DENND4C* rs202246180 | 3.1×10^-8^ |  |  |  |  |  |
| *DFNB31*  rs74551598 | 1.6×10^-8^ |  |  |  |  |  |
| *OIT3*  rs57176252 |  |  | 1.2×10^-8^ |  |  |  |
| *RP11-159H3.2* rs7079858 |  | 4.9×10^-8^ |  |  |  |  |
| *NAP1L4*  rs7935422 |  | 1.5×10^-8^ |  |  |  |  |
| *TRIM5*  rs11601507 |  |  | 6.6×10^-9^ |  |  |  |
| *MACROD1*  rs11231698 |  |  |  | 5.9×10^-9^ |  |  |
| **Sabatti et al (2009)** [[35](#_ENREF_35)] | | | | | | |
| *APOB*  rs673548 |  |  |  | 2.01×10^-8^ | Northern Finland Birth Cohort  n = 4,763 | 31 |
| *GCKR*  rs1260326 |  |  |  | 3.56×10^-10^ |  |  |
| *LPL*  rs10096633 |  |  |  | 5.16×10^-8^ |  |  |
| Unknown  rs2624265 |  |  |  | 4.31×10^-7^ |  |  |
| *NR1H3*  rs2167079 |  | 5.13×10^-8^ |  |  |  |  |
| *NR1H3*  rs7120118 |  | 3.57×10^-8^ |  |  |  |  |
| *LIPC*  rs1532085 |  | 1.77×10^-10^ |  |  |  |  |
| *CETP*  rs3764261 |  | 6.97×10^-29^ |  |  |  |  |
| *LCAT*  rs255049 |  | 3.06×10^-8^ |  |  |  |  |
| Unknown  rs9891572 |  | 2.33×10^-7^ |  |  |  |  |
| *CELSR2-PSRC1 SORT1*  rs646776 |  |  | 2.19×10^-12^ |  |  |  |
| *CR1L*  rs4844614 |  |  | 2.38×10^-7^ |  |  |  |
| *APOB*  rs693 |  |  | 2.99×10^-11^ |  |  |  |
| *FADS1-FADS2* rs174537 |  |  | 2.10×10^-7^ |  |  |  |
| *FADS1-FADS2* rs102275 |  |  | 1.52×10^-7^ |  |  |  |
| *FADS1-FADS2* rs174546 |  |  | 1.30×10^-7^ |  |  |  |
| *FADS1-FADS2* rs174556 |  |  | 3.49×10^-7^ |  |  |  |
| *FADS1-FADS2*  rs1535 |  |  | 3.65×10^-7^ |  |  |  |
| *LDLR*  rs11668477 |  |  | 1.51×10^-7^ |  |  |  |
| *APO cluster*  rs157580 |  |  | 4.96×10^-8^ |  |  |  |
| *AR*  rs5031002 |  |  | 2.37×10^-7^ |  |  |  |
| *ANGPTL3-DOCK7-ATG4C*  rs1167998 |  |  |  | 1.60×10^-4^ |  |  |
| *ANGPTL3-DOCK7-ATG4C*  rs12130333 |  |  |  | 3.60×10^-3^ |  |  |
| *GALNT2*  rs4846914 |  |  |  | 2.80×10^-1^ |  |  |
| *APOB*  rs693 |  |  |  | 3.40×10^-3^ |  |  |
| *GCKR*  rs780094 |  |  |  | 5.03×10^-9^ |  |  |
| *BCL7B-TBL2-MLXIPL*  rs17145738 |  |  |  | 2.70×10^-5^ |  |  |
| *LPL*  rs328 |  |  |  | 4.50×10^-8^ |  |  |
| *TRIB1*  rs6982636 |  |  |  | 1.43×10^-2^ |  |  |
| *APOA1/C3/A4/A5, ZNF259, BUD13* rs12292921 |  |  |  | 1.35×10^-3^ |  |  |
| *LIPC*  rs4775041 |  |  |  | 5.85×10^-1^ |  |  |
| *NCAN-CILP2-PBX4* rs16996148 |  |  |  | 8.40×10^-1^ |  |  |
| *GALNT2*  rs4846914 |  | 5.60×10^-4^ |  |  |  |  |
| *LPL*  rs328 |  | 7.18×10^-6^ |  |  |  |  |
| *ABCA1*  rs2740491 |  | 3.12×10^-4^ |  |  |  |  |
| *ABCA1*  rs3847303 |  | 3.22×10^-3^ |  |  |  |  |
| *APOA1/C3/A4/A5,*  *ZNF259, BUD13*  rs28927680 |  | Not given |  |  |  |  |
| *MVK-MMAB*  rs2338104 |  | 4.40×10^-2^ |  |  |  |  |
| *LIPC*  rs4775041 |  | 1.70×10^-2^ |  |  |  |  |
| *LIPC*  rs1800588 |  | 4.11×10^-3^ |  |  |  |  |
| *CETP*  rs3764261 |  | 6.97×10^-29^ |  |  |  |  |
| *CETP*  rs1800775 |  | 1.32×10^-9^ |  |  |  |  |
| *LCAT*  rs255052 |  | 2.36×10^-7^ |  |  |  |  |
| *LIPG-ACAA2*  rs2156552 |  | 1.15×10^-2^ |  |  |  |  |
| *PCSK9*  rs12117661 |  |  | 1.95×10^-12^ |  |  |  |
| *CELSR2-PSRC1- SORT1*  rs646776 |  |  | 2.19×10^-12^ |  |  |  |
| *APOB*  rs693 |  |  | 2.99×10^-11^ |  |  |  |
| *APOB*  rs562338 |  |  | 1.97×10^-7^ |  |  |  |
| *HMGCR*  rs12654264 |  |  | 2.63×10^-5^ |  |  |  |
| *B3GALT4*  rs2254287 |  |  | 6.98×10^-1^ |  |  |  |
| *LDLR*  rs6511720 |  |  | 1.48×10^-9^ |  |  |  |
| *NCAN-CILP2-PBX4*  rs16996148 |  |  | 8.20×10^-1^ |  |  |  |
| *APO cluster*  rs2075650 |  |  | 1.05×10^-5^ |  |  |  |
| **Heid et al. (2008)** [[36](#_ENREF_36)] | | | | | | |
| *CETP*  rs1800775 |  | 6.05×10^-15^ |  |  | German  n=1643 | 33 – 93 |
| *PRICKLX10* rs17569297 |  | 1.51×10^-6^ |  |  |  |  |
| *CETP*  rs9989419 |  | 1.86×10^-6^ |  |  |  |  |
| *PRICKLX10* rs10506210 |  | 2.22×10^-6^ |  |  |  |  |
| *UBXD2*  rs16831992 |  | 2.44×10^-6^ |  |  |  |  |
| *CACNA2D4* rs11062008 |  | 3.73×10^-6^ |  |  |  |  |
| *SORCS2*  rs734526 |  | 5.34×10^-6^ |  |  |  |  |
| *SHB*  rs10973646 |  | 6.32×10^-6^ |  |  |  |  |
| *PBX1*  rs4657374 |  | 1.04×10^-5^ |  |  |  |  |
| *DKFZP434C171* rs248426 |  | 1.06×10^-5^ |  |  |  |  |
| *ENST0000027724*4 rs1970528 |  | 1.08×10^-5^ |  |  |  |  |
| *NUP160*  rs6485788 |  | 1.22×10^-5^ |  |  |  |  |
| *EDARADD*  rs600988 |  | 1.29×10^-5^ |  |  |  |  |
| *EDARADD*  rs660351 |  | 1.41×10^-5^ |  |  |  |  |
| *ITGB8*  rs11973964 |  | 1.59×^-5^ |  |  |  |  |
| *EDARADD*  rs585537 |  | 1.63×^-5^ |  |  |  |  |
| *SCARB1*  rs12831105 |  | 1.72×^-5^ |  |  |  |  |
| *DGKH*  rs10492434 |  | 1.97×^-5^ |  |  |  |  |
| *ITGB8*  rs6973059 |  | 2.21×^-5^ |  |  |  |  |
| *ITGKB*  rs3768371 |  | 2.22×^-5^ |  |  |  |  |
| *NCAM1*  rs7111410 |  | 2.31×10^-5^ |  |  |  |  |
| *LPL*  rs17482753 |  | 2.71×10^-5^ |  |  |  |  |
| *FZD10*  rs1532724 |  | 2.83×10^-5^ |  |  |  |  |
| *ENST00000357331* rs2526100 |  | 3.03×10^-5^ |  |  |  |  |
| *NCAM1*  rs4936266 |  | 3.13×10^-5^ |  |  |  |  |
| *EDARADD*  rs2463198 |  | 3.22×10^-5^ |  |  |  |  |
| *SYNE1*  rs11752725 |  | 3.26×10^-5^ |  |  |  |  |
| *CTNNA2*  rs1861700 |  | 3.56×10^-5^ |  |  |  |  |
| *LPL*  rs1919484 |  | 3.79×10^-5^ |  |  |  |  |
| *PBX1*  rs3767364 |  | 3.83×10^-5^ |  |  |  |  |
| *ITGB8*  rs6954502 |  | 3.91×10^-5^ |  |  |  |  |
| *TNNI3*  rs2288529 |  | 4.58×10^-5^ |  |  |  |  |
| *CNDP1*  rs12961730 |  | 4.62×10^-5^ |  |  |  |  |
| *SCARB1*  rs11615630 |  | 4.79×10^-5^ |  |  |  |  |
| *NCAM1*  rs2846915 |  | 5.37×10^-5^ |  |  |  |  |
| *ENST00000357331* rs12699252 |  | 5.39×10^-5^ |  |  |  |  |
| *LPL*  rs7461111 |  | 5.60×10^-5^ |  |  |  |  |
| *ITGB8*  rs17365098 |  | 5.80×10^-5^ |  |  |  |  |
| *PARVG*  rs139265 |  | 5.89×10^-5^ |  |  |  |  |
| *TYRP1*  rs7863023 |  | 5.97×10^-5^ |  |  |  |  |
| *NFYA*  rs7760860 |  | 6.07×10^-5^ |  |  |  |  |
| *LPL*  rs17411024 |  | 6.66×10^-5^ |  |  |  |  |
| *NRXN3*  *rs766024* |  | 7.03×10^-5^ |  |  |  |  |
| *SORCS2*  rs16840358 |  | 7.45×10^-5^ |  |  |  |  |
| *LOC387882* rs11112741 |  | 7.89×10^-5^ |  |  |  |  |
| *C14orf147*  rs10130824 |  | 8.03×10^-5^ |  |  |  |  |
| *LPL*  rs1837842 |  | 8.55×10^-5^ |  |  |  |  |
| *CRYBA4*  rs5761635 |  | 9.04×10^-5^ |  |  |  |  |
| *CDKL2*  rs6851864 |  | 9.12×10^-5^ |  |  |  |  |
| *ENST00000078131* rs2478884 |  | 9.12×10^-5^ |  |  |  |  |
| *PBX1*  rs3767368 |  | 9.37×10^-5^ |  |  |  |  |
| *CNDP1*  rs9319909 |  | 9.59×10^-5^ |  |  |  |  |
| *SHB*  rs943938 |  | 9.61×10^-5^ |  |  |  |  |
| *SYT5*  rs12461195 |  | 9.94×10^-5^ |  |  |  |  |
| *LPL*  rs17411126 |  | 1.67×10^-4^ |  |  |  |  |
| *LPL*  rs17489268 |  | 2.01×10^-4^ |  |  |  |  |
| *LPL*  rs271 |  | 2.30×10^-4^ |  |  |  |  |
| *LPL*  rs10503669 |  | 2.58×10^-4^ |  |  |  |  |
| *LPL*  rs17411031 |  | 3.07×10^-4^ |  |  |  |  |
| *LIPG*  rs7240405 |  | 3.19×10^-4^ |  |  |  |  |
| *LIPG*  rs2156552 |  | 3.71×10^-4^ |  |  |  |  |
| *LIPG*  rs1943981 |  | 4.41×10^-4^ |  |  |  |  |
| *LIPG*  rs4939883 |  | 8.45×10^-4^ |  |  |  |  |
| **Saxena et al. (2007)**^c^ [[37](#_ENREF_37)] | | | | | | |
| *GCKR*  rs780094 |  |  |  | 3.7×10^−8^ | Finnish and Swedish  T2D: n = 1,464  Controls: n = 1,467 | Not given |
| *GCKR*  rs780094 |  |  |  | 8.7×10^−8^ |  |  |
| *APOE* cluster rs4420638 |  |  | 3.4×10^−13^ |  |  |  |
| *APOB*  rs693 |  |  | 7.1×10^−7^ |  |  |  |
| *CETP*  rs1800775 |  | 2.5×10^−13^ |  |  |  |  |
| *LPL*  rs17482753 |  | 3.6×10^−5^ |  |  |  |  |
| *LIPC*  rs261332 |  | 3.4×10^−5^ |  |  |  |  |
| *LPL*  rs17482753 |  |  |  | 4.9×10^−7^ |  |  |
| *APOA5*  rs481843 |  |  |  | 3.3×10^−5^ |  |  |
| **Sandhu et al. (2008)**^c^ [[96](#_ENREF_96)] | | | | | | |
| *APOC1*  rs4420638 |  |  | 1.2×10^-2^⁰ |  | White European  (British, Swedish, Finnish and Italian)  n = 11,685 | 39–79 |
| *PSRC1*  rs599839 |  |  | 1.7×10^-1^⁵ |  |  |  |
| *CELSR2*  rs4970834 |  |  | 3.0×10^-11^ |  |  |  |
| *APOB*  rs562338 |  |  | 1.4×10^-^⁹ |  |  |  |
| *APOB*  rs7575840 |  |  | 1.9×10^-^⁹ |  |  |  |
| *APOB*  rs478442 |  |  | 8.1×10^-^⁹ |  |  |  |
| *APOB*  rs4591370 |  |  | 8.2×10^-^⁹ |  |  |  |
| *APOB*  rs4560142 |  |  | 8.3×10^-^⁹ |  |  |  |
| *APOB*  rs576203 |  |  | 9.0×10^-^⁹ |  |  |  |
| *APOB*  rs506585 |  |  | 1.0×10^-^⁸ |  |  |  |
| *APOB*  rs488507 |  |  | 2.0×10^-^⁸ |  |  |  |
| *APOB*  rs538928 |  |  | 2.7×10^-^⁸ |  |  |  |
| *BCAM*  rs10402271 |  |  | 4.1×10^-^⁸ |  |  |  |
| *APOB*  rs693 |  |  | 4.4×10^-^⁸ |  |  |  |
| *TOMM40*  rs2075650 |  |  | 7.1×10^-14^ |  | British Birth Cohort  n = 4,337 | 39 - 79 |
| *BCL3*  rs4803750 |  |  | 2.4×10^-11^ |  |  |  |
| *CELSR2*  rs646776 |  |  | 4.3×10^-^⁹ |  |  |  |
| *APOB*  rs1713222 |  |  | 1.0×10^-^⁸ |  |  |  |
| *LDLR*  rs2228671 |  |  | 1.1×10^-^⁸ |  |  |  |
| *LDLR*  rs11668477 |  |  | 1.5×10^-^⁸ |  |  |  |
| *BCAM*  rs4605275 |  |  | 4.7×10^-^⁸ |  |  |  |
| *CELSR2*  rs646776 |  |  | 3.0×10^-14^ |  | White European  (British, Swedish, Finnish and Italian)  n = 9,988 | 39–79 |
| *CELSR2*  rs629301 |  |  | 3.1×10^-14^ |  |  |  |
| *CELSR2*  rs12740374 |  |  | 3.2×10^-14^ |  |  |  |
| *CELSR2*  rs660240 |  |  | 3.8×10^-14^ |  |  |  |
| *CELSR2*  rs602633 |  |  | 5.7×10^-14^ |  |  |  |
| *CELSR2*  rs599839 |  |  | 7.8×10^-11^ |  |  |  |
| *CELSR2*  rs611917 |  |  | 1.5×10^-10^ |  |  |  |
| *CELSR2*  rs4970834 |  |  | 6.7×10^-10^ |  |  |  |
| *CELSR2*  rs6657811 |  |  | 2.0×10^-8^ |  |  |  |
| **Surakka et al (2012)** [[97](#_ENREF_97)] | | | | | | |
| *SRGAP2*  rs2483058 |  | 3.98×10^-8^ |  |  | European  (Monozygotic women twin-pairs)  n = 1,720 | 20 - 80 |
| *SRGAP2*  rs9242 |  | 1.08×10^-7^ |  |  |  |  |
| *CD47*  rs17826288 |  |  |  | 8.16×10^-7^ |  |  |
| **Wallace et al (2008)** [[41](#_ENREF_41)] | | | | | | |
| *ApoA5*  rs6589567 | 7.76×10^-7^ |  |  |  | British  (Hypertensive)  n = 1,955 | Median = 58  Interquartile range 49 - 65 |
| *CELSR2*  rs4970834 | 1.70×10^-6^ |  |  |  |  |  |
| Unknown  rs10514542 | 6.98×10^-6^ |  |  |  |  |  |
| *TBPL2*  rs4470077 | 9.04×10^-6^ |  |  |  |  |  |
| Unknown  rs11017236 |  | 5.67 × 10^-7^ |  |  |  |  |
| Unknown  rs11826048 |  | 9.70 × 10^-7^ |  |  |  |  |
| *COLQ*  rs905648 |  | 4.58 × 10^-6^ |  |  |  |  |
| *PSRC1, CELSR2* rs599839 |  |  | 1.05 × 10^-7^ |  |  |  |
| Unknown  rs11889082 |  |  | 1.22×10^-6^ |  |  |  |
| *NA*  rs6470600 |  |  | 8.68×10^-6^ |  |  |  |
| *APOA5*  rs6589566 |  |  |  | 2.89×10^-11^ |  |  |
| *LPL*  rs17482753 |  |  |  | 1.17×10^-9^ |  |  |
| *GCKR*  rs780094 |  |  |  | 4.99×10^-7^ |  |  |
| Unknown  rs17545624 |  |  |  | 2.13×10^-6^ |  |  |
| **Weissglas-Volkov et al (2013)** [[38](#_ENREF_38)] | | | | | | |
| *APOA5*  rs964184 |  |  |  | 5.5×10^-35^ | Mexican  n = 2,240 | Not given |
| *GCKR*  rs1260326 |  |  |  | 2.2×10^-13^ |  |  |
| *LPL*  rs12678919 |  |  |  | 2.7×10^-10^ |  |  |
| *MLXIPL*  rs2286276 |  |  |  | 2.2×10^-6^ |  |  |
| *TIMD4*  rs2036402 |  |  |  | 3.4×10^-6^ |  |  |
| *CILP2*  rs2228603 |  |  |  | 3.0×10^-5^ |  |  |
| *ANGPTL3*  rs10889337 |  |  |  | 3.3×10^-5^ |  |  |
| *CETP*  rs1532624 |  | 1.39×10^-24^ |  |  |  |  |
| *LIPC*  rs1077835 |  | 2.1×10^-14^ |  |  |  |  |
| *LOC55908*  rs2278426 |  | 3.44×10^-9^ |  |  |  |  |
| *ABCA1*  rs9282541 |  | 6.4×10^-26^ |  |  |  |  |
| Unknown  rs4149310 |  | 5.54×10^-8^ |  |  |  |  |
| ^a^ P-values are for the discovery stage of the genome-wide association study.  ^b^ For meta-analysis where the combined p-value is provided, this has been given in the table.  ^c^ Meta-analysis  ^d^ Lowest mean age and highest mean age.  ^e^ P values are expressed as -Log10P  SNP ̶ single-nucleotide polymorphism; TC ̶ total cholesterol; HDL ̶ high-density lipoprotein cholesterol; LDL ̶ low-density lipoprotein cholesterol; TG ̶ triglycerides; T2D ̶ type 2 diabetes. | | | | | | |

Supplemental Table 2. Observational Studies Examining Interaction Between *CETP* Polymorphisms and Diet on Blood Lipids

| SNP (Nucleotide Change)^a^ | Minor Allele/  Effect Allele | Ethnicity & Sample Size | Age  (Years) | Dietary Factor | Design | Lipid Trait Examined | Results for Interaction^b^ | References |
| --- | --- | --- | --- | --- | --- | --- | --- | --- |
| TaqIB (rs708272)  (G > A) | A/A | Mexican - Mestizo  n = 215 | 36.9 ± 11.7 | Energy, proteins, fat, SFA, MUFA, PUFA, ratio n-6:n-3, carbohydrates, simple carbohydrates,  sucrose, cholesterol and fiber. | Cross-sectional; three – day food intake record. | TC, HDL, LDL and TG | Among participants with a higher intake of sucrose (≥ 5% of the total kcal/day), those carrying the minor allele (A) had higher TC (mean TC (mg/dl): 200.19 vs 165.55, P_interaction_ = 0.034) and higher LDL (mean LDL (mg/dl): 128.64 vs 99.29, P_interaction_ = 0.037) compared with those carrying 2 copies of the major allele (G).  None of the other SNP-diet interactions were statistically significant. | Campos-Perez et al.  (2020) [[47](#_ENREF_47)] |
| TaqIB (rs708272)  (G > A) | A/G | Iranian  patients with T2D without dyslipedaemia: n=129  Patients with T2D and dyslipedemia:  n = 55 | 52.9 ± 0.6 | Energy, total fat, PUFA, MUFA, cholesterol, carbohydrate, fiber and alcohol intake. | Cross-sectional; food frequency questionnaire. | HDL | Among participants without dyslipidaemia, a higher intake of total fat (>34.9 % from total energy intake) was associated with higher HDL in individuals with 2 copies of the major allele (G) compared with those carrying 2 copies of the minor allele (A) (mean HDL (mg/dl) for high total fat vs low total fat intake (≤34.9% from total energy) in ‘GG’: 58.6 ± 4.1 vs 36.5 ± 6.5; P_interaction_ = 0.02).  None of the other SNP-diet interactions were statistically significant. | Kalantar et al. (2018) [[15](#_ENREF_15)] |
| TaqIB (rs708272)  (G > A) | A/ | Spanish  Patients with T2D, obesity, hypertension or dyslipedaemia:  n = 4210 | 66.9 ± 6.3 | Energy,  total fat, SFA, MUFA, PUFA, proteins, carbohydrates and alcohol intake. | Nested case-control  Food frequency questionnaire. | TC, HDL, LDL and TG. | None of the SNP-diet interactions were statistically significant. | Corella et al. (2010b) [[57](#_ENREF_57)] |
| TaqIB (rs708272)  (G > A) | A/ | Spanish  patients with CHD:  n = 557  Healthy controls:  n = 1180 | 53.9 ± 7.3 | Alcohol consumption. | Nested case-control; validated computerised diet history questionnaire. | TC, HDL, LDL and TG | None of the SNP-diet interactions were statistically significant. | Corella et al. (2010a) [[56](#_ENREF_56)] |
| TaqIB (rs708272)  (G > A) | A/G | Men with T2D without CVD in the USA  (96% white participants)  n = 603 | 40 - 75 | Energy, total fat, animal fat, vegetable fat, cholesterol, PUFA, MUFA, trans fat, SFA and alcohol intake. | Prospective cohort; food-frequency questionnaire. | HDL, LDL, TG and non-HDL cholesterol. | A higher intake of total fat (>33.5% from total energy intake), animal fat (>19.9% from total energy intake), SFA (>11.47% from total energy intake) and MUFA (>12.75% from total energy intake) was associated with lower HDL in participants with 2 copies of the major allele (G) compared with those with AA genotype (Pint_eraction_ = 0.003, 0.02, 0.02 and 0.04 respectively).  None of the other SNP-diet interactions were statistically significant. | Li et al. (2007)[[13](#_ENREF_13)] |
| TaqIB (rs708272)  (G > A) | A/A | Multi-ethnic  Chinese: n = 2858  Malay: n = 761  Asian Indian:  n = 588 | 37.2^c^ – 41.1^c^ | Energy, percentage of energy as fat and cholesterol intake. | Cross-sectional; food frequency questionnaire. | TC, HDL, LDL and TG | In Malay and Asian Indian participants, a higher dietary cholesterol (cholesterol intake as continuous) was linked to higher HDL in participants with 2 copies of the A allele compared with those with GG genotype (P_interaction_ = 0.046 for Malay; P_interaction_ = 0.023 for Indian). The interaction was not statistically significant in Chinese.  None of the other SNP-diet interactions were statistically significant. | Tai et al. (2003) [[17](#_ENREF_17)] |
| TaqIB (rs708272)  (G > A) | A/A | Irish and French  Male MI patients:  n = 608  Healthy controls:  n = 724 | 52.7^c^ - 53.7^c^ | Alcohol consumption. | Case-control; questionnaire. | HDL, LDL, TG and VLDL | Among healthy controls, a higher intake of alcohol (≥ 75g /day) was associated with higher levels of HDL in participants carrying the minor allele (A) (30% higher for ‘AA’ and 13% higher for ‘GA’) compared with those with GG genotype (P_interaction_ < 0.0001).  None of the other SNP-diet interactions were statistically significant. | Fumeron et al. (1995) [[51](#_ENREF_51)] |
| TaqIB (rs708272)  (G > A) | A/A | Multi-ethnic  (11 US States)  CHD patients: n = 505  Healthy controls:  n = 999 | 62^c^ – 66^c^ | Alcohol consumption. | Nested case-control; food frequency questionnaire. | TC, HDL, LDL and  TG | In healthy controls, a higher alcohol intake (≥15 g/day) was linked to higher HDL in participants carrying the minor allele (A), with those carrying 2 copies having the highest HDL (P_interaction_ < 0.01).  None of the other SNP-diet interactions were statistically significant. | Jensen et al. (2008) [[49](#_ENREF_49)] |
| TaqIB (rs708272)  (G > A) | A/A | Japanese  (Western Japan)  n = 1729 | 57.2 ± 15.7 | Alcohol consumption. | Cross-sectional; questionnaire. | TC, HDL and TG | A higher alcohol intake (≥ 2 drinks/day) was associated with higher HDL in men carrying the minor allele (A) compared with men carrying 2 copies of the major allele (G) (mean HDL (mmol/l): ‘GG’, 1.37 ± 0.03; ‘GA’, 1.44 ± 0.03; ‘AA’, 1.49 ± 0.05; P_interaction_ = 0.049). In women, consumption of any amount of alcohol was linked to higher HDL in individuals with 2 copies of the A allele compared with those with GG or GA genotypes (mean HDL (mmol/l): ‘GG’, 1.57±0.03; ‘GA’, 1.57±0.03; ‘AA’, 1.79±0.06; P_interaction_ = 0.022).  None of the other SNP-diet interactions were statistically significant | Tsujita et al. (2007) [[50](#_ENREF_50)] |
| TaqIB (rs708272)  (G > A) | A/G and A | Hei Yi Zhuang Chinese  Healthy participants  n = 758 | 39.9^c^ – 42.4^c^ | Alcohol consumption. | Cross-sectional; questionnaire. | TC, HDL, LDL and TG | In participants with 2 copies of the major allele (G), those who consumed any amount of alcohol had higher HDL (mean HDL (mmol/l): 2.09 ± 0.46 vs 1.94 ± 0.38; P< 0.01) and TG (mean TG (mmol/l): 1.42 ± 2.71 vs 0.94 ± 0.36; P< 0.05 ) and lower LDL (mean LDL (mmol/l): 2.24 ± 0.65 vs 2.65 ± 3.01; P < 0.01) compared with those who did not drink alcohol.  In heterozygotes (GA), HDL was higher in drinkers than non-drinkers (mean HDL (mmol/l): 2.17 ± 0.55 vs 2.02 ± 0.50; P < 0.05). Those with GA genotype who drank any amount of alcohol also had lower TG than individuals with GG who drank any amount of alcohol (mean TG (mmol/l): 1.01 ± 0.86 vs 1.42 ± 2.71; P < 0.05).  None of the other SNP-diet interactions were statistically significant. | Zhou et al. (2008) [[48](#_ENREF_48)] |
| TaqIB (rs708272)  (G > A) | A/ | Scottish  Healthy participants  n = 220 | 39 ± 11 | Alcohol consumption. | Cross-sectional; questionnaire. | TC, HDL, LDL, VLDL and TG | None of the SNP-diet interactions were statistically significant. | Freeman et al. (1994) [[55](#_ENREF_55)] |
| TaqIB (rs708272)  (G > A) | A/ | White American and African American  n = 15,792 | 53.7 ± 0.5 | Alcohol consumption. | Longitudinal; dietary questionnaire. | TC and HDL | None of the SNP-diet interactions were statistically significant. | Volcik et al. (2007) [[54](#_ENREF_54)] |
| TaqIB (rs708272)  (G > A) | A/ | White British  Healthy men  n = 2773 | 56 ± 3.4 | Alcohol consumption. | Longitudinal; questionnaire. | TC, HDL and TG | None of the SNP-diet interactions were statistically significant. | Talmud et al. (2002) [[53](#_ENREF_53)] |
| TaqIB (rs708272)  (G > A) | A/ | Inuit  (Nunavik Inuit)  n = 553 | 37.2 ± 8.5 | n-3 PUFA in red blood cells (RBCs) and total energy intake. | Cross-sectional: gas chromatographic analysis; food frequency questionnaire. | TC, HDL, LDL and TG. | None of the SNP-diet interactions were statistically significant. | Rudkowska et al. (2013b) [[71](#_ENREF_71)] |
| TaqIB (rs708272)  (G > A) | A/ | Inuit  (Nunavik Inuit)  n = 553 | 37.2 ± 8.5 | Total energy, total fat and total SFA intake. | Cross-sectional; food frequency questionnaire. | TC, HDL,  LDL and TG | None of the SNP-diet interactions were statistically significant. | Rudkowska et al. (2013a) [[16](#_ENREF_16)] |
| TaqIB (rs708272)  (G > A) | A/ | White American  n = 8,968  African American  n = 2, 677 | 53.6 ± 0.5 | Energy, protein, carbohydrate, total fat, SFA, MUFA, PUFA, cholesterol and fiber intake. | Cross-sectional; food frequency questionnaire. | TC, HDL, LDL and TG | None of the SNP-diet interactions were statistically significant. | Nettleton et al. (2007) [[98](#_ENREF_98)] |
| TaqIB (rs708272)  (G > A) | A/ | Finnish  Male alcohol drinkers:  n = 98  Male healthy non-alcoholic controls:  n = 82 | 41.6 ± 9.7 | Alcohol consumption. | Cross-sectional; interview. | TC, HDL, LDL, VLDL, TG and VLDL-TG | None of the SNP-diet interactions were statistically significant. | Hannuksela et al. (1994) [[52](#_ENREF_52)] |
| rs5882 (I405V)  (G > A) | G/G | Iranian  n = 4700 | 40.9 ± 13.9 | Energy, carbohydrate, protein, total fat, MUFA, PUFA, SFA, fish and fiber intake. | Longitudinal: 3.6 years of follow-up; food frequency questionnaire. | TC, HDL, LDL and TG | Higher total fat intake was associated with increased levels of TG in participants carrying the minor allele (G) compared to those carrying 2 copies of the major (A) allele (mean changes in TG (mg/dl) across quartiles of total fat intake: −1.90, 2.6, 6.06, 8.88; P_interaction_ = 0.001).  Higher MUFA intake was also linked to increased levels of TG in G allele carriers compared to participants with AA genotype (mean changes in TG (mg/dl) across quartiles of MUFA intake: −3.03, 1.73, 8.06, 8.85; P_interaction_ = 0.001); while higher carbohydrate intake correlated with decreased levels of TG in those carrying the G allele (changes in TG (mg/dl) across quartiles of carbohydrate intake: 6.65, 7.29, 4.42, −3.28; P_interaction_ = 0.01).  None of the other SNP-diet interactions were statistically significant. | Hosseini-Esfahani et al. (2019) [[65](#_ENREF_65)] |
| rs5882 (I405V)  (G > A) | G/G | Iranian  Participants with Metabolic Syndrome (MetS): n = 441  Healthy controls:  n = 844 | 36.9 ± 10.5 | Energy, carbohydrate, protein, total fat, PUFA, MUFA, trans-fatty acids, cholesterol and omega3 fatty acids. | Nested case-control: 3 years of follow-up; food frequency questionnaire. | HDL and TG | Minor allele (G) carriers had a lower risk of low HDL with a lower intake of MUFA and a higher risk of low HDL with a higher intake of MUFA (9.6-11% of total energy intake) compared to participants with AA genotype (P_interaction_ = 0.02).  None of the other SNP-diet interactions were statistically significant. | Esfandiar et al. (2018) [[66](#_ENREF_66)] |
| rs5882 (I405V)  (G > A) | G/G | Multi-ethnic  (USA)  n=101 | 36.2 ± 5.8 | Energy, total fat, SFA, MUFA and PUFA intake. | Cross-sectional analysis from an ongoing clinical trial (NCT02740439)7-day food record. | TC, HDL and TG | A higher MUFA intake (>31g/day) was linked to lower TG in participants carrying the minor allele (G) (P_interaction_ = 0.006).  None of the other SNP-diet interactions were statistically significant. | Hannon et al. (2020) [[10](#_ENREF_10)] |
| rs5882 (I405V)  (G > A) | G/A | Inuit  (Nunavik Inuit)  n = 553 | 37.2 ± 8.5 | n-3 PUFA in red blood cells (RBCs) and total energy intake. | Cross-sectional: gas chromatographic analysis; food frequency questionnaire. | TC, HDL,  LDL and TG. | A higher level of n-3 PUFA in RBCs was associated with lower TC in participants with 2 copies of the major allele (A) compared to those with ‘GG’ or ‘AG’ genotype (β (mmol/l) = -0.0290 ± 0.0307; P_interaction_ = 0.0334) and higher HDL in carriers of the A allele compared to those with ‘GG’ genotype (β (mmol/l) = 0.0263 ± 0.0115 for ‘AG’ genotype, β (mmol/l) = 0.0017 ± 0.0131 for ‘AA’ genotype; P_interaction_ = 0.0271).  None of the other SNP-diet interactions were statistically significant. | Rudkowska et al. (2013b) [[71](#_ENREF_71)] |
| rs5882 (I405V)  (G > A) | G/A | Inuit  (Nunavik Inuit)  n = 553 | 37.2 ± 8.5 | Total energy, total fat and SFA intake. | Cross-sectional; food frequency questionnaire. | TC, HDL,  LDL and TG | In participants with 2 copies of the major allele (A), a higher total fat intake resulted in a greater increase in TC compared with participants with ‘GG’ or ‘AG’ genotype (β (mmol/l) = 0·0024 ± 0·0026; P_interaction_ = 0.046).  None of the other SNP-diet interactions were statistically significant. | Rudkowska et al. (2013a) [[16](#_ENREF_16)] |
| rs5882 (I405V)  (G > A) | G/ | Spanish  n = 1315 | 49.7 ± 0.2 | Plasma selenium | Cross-sectional; inductively coupled-plasma mass spectrometry. | TC, HDL, LDL and TG | None of the SNP-diet interactions were statistically significant. | Galan-Chilet et al. (2015) [[86](#_ENREF_86)] |
| rs5882 (I405V)  (G > A) | G/ | Irish and French  Male participants  MI patients: n = 568  Healthy controls:  n = 668 | 53.2 ± 8.5 | Alcohol consumption. | Case-control; questionnaire. | HDL | None of the SNP-diet interactions were statistically significant. | Corbex et al. (2000) [[82](#_ENREF_82)] |
| rs5882 (I405V)  (G > A) | G/G | Icelandic  Healthy participants  Men: n = 152  Women: n = 166 | 15 - 78 | Alcohol consumption. | Cross-sectional; questionnaire. | HDL and TG | In men, alcohol consumption was associated with higher HDL (13.7% higher HDL than ‘AA’ genotype) in carriers of 2 copies of the minor allele (G) compared with men with AG and AA genotypes (P_interaction_ = 0.026).  None of the other SNP-diet interactions were statistically significant. | Gudnason et al. (1997) [[70](#_ENREF_70)] |
| rs3764261  (C > A) | A/A | Iranian  n = 4,700 | 40.9 ± 13.9 | Energy, carbohydrate, protein, total fat, MUFA,  PUFA, SFA, fish and fiber intake. | Longitudinal: 3.6 years of follow-up; food frequency questionnaire. | TC, HDL, LDL and TG | A higher fish intake was associated with a larger decrease in TC in participants carrying the minor allele (A) (mean changes in TC (mg/dl) with quartiles of fish intake: 8.02, 6.93, 6.54, 5.58) compared to those with CC genotype (mean changes in TC (mg/dl) with quartiles of fish intake: 3.65, 6.62, 4.57, 8.93) (P_interaction_ = 0.02).  None of the other SNP-diet interactions were statistically significant. | Hosseini-Esfahani et al. (2019) [[65](#_ENREF_65)] |
| rs3764261  (C > A) | A/ | Iranian  Participants with MetS:  n = 441  Healthy controls:  n = 844 | 36.9 ± 10.5 | Energy, carbohydrate, protein, total fat, PUFA, MUFA, trans-fatty acids, cholesterol and omega3 fatty acids. | Nested case-control: 3 years of follow-up; food frequency questionnaire. | HDL and TG | None of the SNP-diet interactions were statistically significant. | Esfandiar et al. (2018) [[66](#_ENREF_66)] |
| rs3764261  (C > A) | A/A | Indian  (Lucknow, Nagpur, Hyderabad and Bangalore)  n = 3342 | 39.9 ± 10.3 | Average daily fat intake | Cross-sectional; food frequency questionnaire. | TC, HDL, LDL and TG | Participants carrying the minor allele (A) who had a higher dietary fat intake (≥76.98g/day) had increased levels of TC (β (mmol/l) = 0.097 ± 0.041; P_interaction_ = 0.018) and LDL (β (mmol/l) = 0.085 ± 0.041; P_interaction_ = 0.042).  None of the other SNP-diet interactions were statistically significant. | Walia et al. (2014) [[76](#_ENREF_76)] |
| rs3764261  (C > A) | A/ | White American  Participants with CVD  n = 772 | 66.2 ± 9.4 | Cholesterol and total caloric intake. | Cross-sectional; food frequency questionnaire. | TC | None of the SNP-diet interactions were statistically significant. | Kim et al. (2013) [[99](#_ENREF_99)] |
| C-629A (rs1800775)  (C > A) | C/ | Multi-ethnic  Chinese: n = 1366  Malay: n = 467  Indian: n = 387 | 39.1 ± 12.3 | Cholesterol, energy intake and percentage of energy as fat. | Cross-sectional; food frequency questionnaire. | HDL | None of the SNP-diet interactions were statistically significant. | Tai et al. (2003) [[17](#_ENREF_17)] |
| C-629A (rs1800775)  (C > A) | C/A | Irish and French  Men  MI patients: n = 568  Healthy controls:  n = 668 | 53.2 ± 8.5 | Alcohol consumption. | Case-control; questionnaire. | HDL | Among participants carrying the A allele, alcohol consumption was associated with higher HDL in healthy participants (P_interaction_ < 0.002) and patients who were not treated with lipid-lowering medication (P_interaction_ < 0.001).  None of the other SNP-diet interactions were statistically significant. | Corbex et al. (2000) [[82](#_ENREF_82)] |
| C-629A (rs1800775)  (C > A) | C/ | Inuit  (Nunavik Inuit)  n = 553 | 37.2 ± 8.5 | n-3 PUFA in red blood cells (RBCs) and total energy intake. | Cross-sectional: Gas chromatographic analysis; Food frequency questionnaire. | TC, HDL,  LDL and TG. | None of the SNP-diet interactions were statistically significant. | Rudkowska et al. (2013b) [[71](#_ENREF_71)] |
| C-629A (rs1800775)  (C > A) | C/ | Inuit  (Nunavik Inuit)  n = 553 | 37.2 ± 8.5 | Total energy, total fat and SFA intake. | Cross-sectional, food frequency questionnaire. | TC, HDL,  LDL and TG | None of the SNP-diet interactions were statistically significant. | Rudkowska et al. (2013a) [[16](#_ENREF_16)] |
| C-629A (rs1800775)  (C > A) | C/C | Taiwanese  n = 9075 | 53.5 ± 0.4 | Coffee  consumption. | Cross-sectional; questionnaire. | HDL | Coffee consumption was associated with lower HDL in women carrying the minor allele (C) compared to women with ‘AA’ genotype (β = -1.8095 (standard error not given) for ‘AC’ genotype, β (mg/dl) = -2.8151 for ‘CC’ genotype; P_interaction_ < 0.0001); and in men carrying the ‘C’ allele compared to men with the ‘AA’ genotype [β (mg/dl) = -1.9623 for ‘AC’ genotype, β (mg/dl) = -2.7153 for ‘CC’ genotype; P_interaction_<0.0001]  None of the other SNP-diet interactions were statistically significant. | Hsu et al. (2019) [[81](#_ENREF_81)] |
| C-4502T (rs183130)  (C > T) | T/ | Spanish  Patients with T2D, Obesity, Hypertension or Dyslipedemia.  n = 4210 | 66.9 ± 6.3 | Energy,  total fat, SFA, MUFA, PUFA, protein, carbohydrate and alcohol intake. | Nested case-control  Food frequency questionnaire | TC, HDL, LDL and TG. | None of the SNP-diet interactions were statistically significant. | Corella et al. (2010b) [[57](#_ENREF_57)] |
| C-4502T (rs183130)  (C > T) | T/T and C | Inuit  (Nunavik Inuit)  n = 553 | 37.2 ± 8.5 | n-3 PUFA in red blood cells (RBCs) and total energy intake. | Cross-sectional: gas chromatographic analysis; food frequency questionnaire. | TC, HDL,  LDL and TG. | A higher level of n-3 PUFA in RBCs was associated with lower TC in carriers of the minor allele (T) compared to those with ‘CC’ genotype [β (mmol/l) = −0.0632 ± 0.0241 for CT, β (mmol/l) = −0.0421 ± 0.0343 for TT; P_interaction_ = 0.0326] and lower TG in heterozygotes compared to those with TT genotype [β (mmol/l) = −0.0095 ± 0.0051 vs 0.0073 ± 0.0073; P_interaction_ =0.0300].  None of the other SNP-diet interactions were statistically significant. | Rudkowska et al. (2013b) [[71](#_ENREF_71)] |
| C-4502T (rs183130)  (C > T) | T/ | Inuit  (Nunavik Inuit)  n = 553 | 37.2 ± 8.5 | Total energy, total fat and SFA intake. | Cross-sectional, food frequency questionnaire. | TC, HDL,  LDL and TG | None of the SNP-diet interactions were statistically significant. | Rudkowska et al. (2013a) [[16](#_ENREF_16)] |
| rs4783961  (G > A) | A/G | Inuit  (Nunavik Inuit)  n = 553 | 37.2 ± 8.5 | n-3 PUFA in red blood cells (RBCs) and total energy intake. | Cross-sectional: gas chromatographic analysis; food frequency questionnaire. | TC, HDL,  LDL and TG. | A higher level of n-3 PUFA in RBCs was associated with lower TG (β (mmol/l) = -0.0106 ± 0.0057; P_interaction_ = 0.0032) and lower TC:HDL ratio (β (mmol/l) = -0.0055 ± 0.0033; P_interaction_ = 0.0483) in heterozygotes compared to participants with 2 copies of the minor allele (A). | Rudkowska et al. (2013b)[[71](#_ENREF_71)] |
| rs4783961  (G > A) | A/ | Inuit  (Nunavik Inuit)  n = 553 | 37.2 ± 8.5 | Total energy, total fat and SFA intake. | Cross-sectional; food frequency questionnaire. | TC, HDL,  LDL and TG | None of the SNP-diet interactions were statistically significant. | Rudkowska et al. (2013a) [[16](#_ENREF_16)] |
| rs9989419  (A > G) | A/ | Swiss  n = 5409 | 53.4 ± 10.8 | Alcohol consumption. | Cross-sectional; reported alcohol consumption of the last 7 days. | TC, HDL and TG. | None of the SNP-diet interactions were statistically significant. | Marques-Vidal et al. (2010) [[100](#_ENREF_100)] |
| rs6499861  (C > G) | G/ | Swiss  n = 5409 | 53.4 ± 10.8 | Alcohol consumption. | Cross-sectional; reported alcohol consumption of the last 7 days. | TC, HDL and TG. | None of the SNP-diet interactions were statistically significant. | Marques-Vidal et al. (2010) [[100](#_ENREF_100)] |
| C>T/In9 (rs289714)  (G > A) | G/A | Multi-ethnic  (USA)  n=101 | 36.2 ± 5.8 | Energy, total fat, SFA, MUFA and PUFA intake. | Cross-sectional analysis from an ongoing clinical trial (NCT02740439)7-day food record. | TC, HDL and TG | Among participants with 2 copies of the major allele (A), those with an intake of >92g of total fat /day had lower TG (103 ± 63 vs 135 ± 15 mg/dl) than those who consumed <31g of total fat /day (P_interaction_ = 0.001).  None of the other SNP-diet interactions were statistically significant. | Hannon et al. (2020) [[10](#_ENREF_10)] |
| rs1800774  (C > T) | T/C | Spanish  n = 1315 | 49.7 ± 0.2 | Plasma selenium | Cross-sectional; inductively coupled-plasma mass spectrometry. | TC, HDL, LDL and TG | Higher plasma selenium levels were associated elevated LDL in all the three genotypes but participants with 2 copies of the major allele (C) had lower LDL compared to those with ‘CT’ and ‘TT’ genotypes [odds ratio per an interquintile range increase in plasma selenium (95% confidence interval): 0.97 (0.74 to 1.27) for ‘CC’, 1.76 (1.38 to 2.25) for ‘CT’, 3.20 (1.93 to 5.28) for ‘TT’ genotype; P_interaction_ = 0.0002].  None of the other SNP-diet interactions were statistically significant. | Galan-Chilet et al. (2015) [[86](#_ENREF_86)] |
| rs4783962  (T > A / T > C) | T/ | Spanish  n = 1315 | 49.7 ± 0.2 | Plasma selenium | Cross-sectional; inductively coupled-plasma mass spectrometry. | TC, HDL, LDL and TG | None of the SNP-diet interactions were statistically significant. | Galan-Chilet et al. (2015) [[86](#_ENREF_86)] |
| rs820299  (G > A / G > C) | G/ | Taiwanese  n = 3000 | 49.2 ± 11.0 | Alcohol consumption. | Cross-sectional; questionnaire. | HDL and TG | None of the SNP-diet interactions were statistically significant. | Lin et al. (2016) [[101](#_ENREF_101)] |
| C373  (Ala > Pro) | Pro/ | Irish and French  Male participants  MI patients:  n = 568  Healthy controls:  n = 668 | 53.2 ± 8.5 | Alcohol consumption. | Case-control; questionnaire. | HDL | None of the SNP-diet interactions were statistically significant. | Corbex et al. (2000) [[82](#_ENREF_82)] |
| C451  (Arg > Gln) | Gln/ | Irish and French  Male participants  MI patients:  n = 568  Healthy controls:  n = 668 | 53.2 ± 8.5 | Alcohol consumption. | Case-control; questionnaire. | HDL | None of the SNP-diet interactions were statistically significant. | Corbex et al. (2000) [[82](#_ENREF_82)] |
| –631  (C > A) | A/ | Irish and French  Male participants  MI patients:  n = 568  Healthy controls:  n = 668 | 53.2 ± 8.5 | Alcohol consumption. | Case-control; questionnaire. | HDL | None of the SNP-diet interactions were statistically significant. | Corbex et al. (2000) [[82](#_ENREF_82)] |
| +524  (G > T) | T/ | Irish and French  Male participants  MI patients:  n = 568  Healthy controls:  n = 668 | 53.2 ± 8.5 | Alcohol consumption. | Case-control; questionnaire. | HDL | None of the SNP-diet interactions were statistically significant. | Corbex et al. (2000) [[82](#_ENREF_82)] |
| rs1532624  (C > A) | A/ | Multi-ethnic  (USA)  n=101 | 36.2 ± 5.8 | Energy, total fat, SFA, MUFA and PUFA intake. | Cross-sectional analysis from an ongoing clinical trial (NCT02740439)7-day food record. | TC, HDL and TG | None of the SNP-diet interactions were statistically significant. | Hannon et al. (2020) [[10](#_ENREF_10)] |
| TaqIA  (A1 > A2) | A2/ | Finnish  Male alcohol drinkers:  n = 98  Male healthy non-alcoholic controls:  n = 82 | 41.6 ± 9.7 | Alcohol consumption. | Cross-sectional; interview. | TC, HDL, LDL, VLDL, TG and VLDL-TG. | None of the SNP-diet interactions were statistically significant. | Hannuksela et al. (1994) [[52](#_ENREF_52)] |
| EcoNI  (N1 > N2) | N2/ | Finnish  Male alcohol drinkers:  n = 98  Male healthy non-alcoholic controls:  n = 82 | 41.6 ± 9.7 | Alcohol consumption. | Cross-sectional; interview. | TC, HDL, LDL, VLDL, TG and VLDL-TG. | None of the SNP-diet interactions were statistically significant. | Hannuksela et al. (1994) [[52](#_ENREF_52)] |
| ^a^ Alleles are reported in the forward direction in line with dbSNP.  ^b^ P_interaction_ values are reported only for the significant SNP-diet interactions.  ^c^ Lowest mean age and highest mean age  SNP ̶ single-nucleotide polymorphism; TC ̶ total cholesterol; HDL ̶ high-density lipoprotein cholesterol; LDL ̶ low-density lipoprotein cholesterol; VLDL ̶ very low-density lipoprotein cholesterol; TG ̶ triglycerides; VLDL-TG ̶ very low-density lipoprotein triglycerides; SFA ̶ saturated fatty acids; PUFA ̶ polyunsaturated fatty acids; MUFA – monounsaturated fatty acids; n-6 – omega 6 polyunsaturated fatty acids; n-3 ̶ omega 3 polyunsaturated fatty acids; T2D ̶ type 2 diabetes; CHD – coronary heart disease; CVD – cardiovascular disease; MI – myocardial infarction. | | | | | | | | |

Supplemental Table 3. Interventional Studies Examining Interaction Between *CETP* Polymorphisms and Diet on Blood Lipids

| SNP (Nucleotide Change)^a^ | Minor Allele/ Effect Allele | Ethnicity & Sample Size | Age (Years) | Dietary Factor | Intervention | Lipid Trait Examined | Results for Interaction^b^ | References |
| --- | --- | --- | --- | --- | --- | --- | --- | --- |
| TaqIB (rs708272)  (G > A) | A/G | Iranian  patients with T2D:  n = 95  Healthy controls:  n = 73 | 48.0 ± 1.7 | Sesame oil, Canola oil and Sesame-canola oil. | Randomised triple-blind crossover trial; Three diets: Sesame oil; canola oil; 40% sesame oil and 60% canola oil. 4-week washout period with sunflower oil; three 9-week intervention periods, separated by 4-week washout periods. | TC, HDL, LDL and TG | In healthy participants, none of the SNP-diet interactions were statistically significant.  In patients with T2D, carriers of 2 copies of the major allele (G) had a reduction in lipid ratios after intake of sesame oil and sesame-canola oil (change in LDL: HDL (mg/dl), -1.29, P_interaction_ = 0.027;  change in TC: HDL (mg/dl), -2.82, P_interaction_ = 0.024; and change in TG: HDL (mg/dl), -7.00; P_interaction_ = 0.025).  None of the other SNP-diet interactions were statistically significant. | Ramezani-Jolfaie et al. (2020) [[61](#_ENREF_61)] |
| TaqIB (rs708272)  (G > A) | A/G | Spanish  Prepubertal children  with mild hypercholesterolemia  n = 36 | 8.4 ± 2.9 | MUFA from virgin olive oil. | Crossover: Cow’s skim milk vs cow’s skim milk enriched with virgin olive oil; 2 periods of 6 weeks. | HDL, LDL and TG. | Intake of olive-oil-enriched skim milk resulted in a higher increase in HDL [mean change in HDL (mmol/) (95% confidence interval) l: 0.179 (0.096 to 0.262) vs 0.089 (0.032 to 0.146); P_interaction_ < 0.001] and a decrease in LDL:HDL ratio [mean change in LDL:HDL (mmol/l) (95% confidence interval): -0.470 (-0.729 to 0.211) vs -0.097 (-0.275 to 0.081); P_interaction_ < 0.001] in participants with 2 copies of the major allele (G) compared with carriers of the minor allele (A).  None of the other SNP-diet interactions were statistically significant. | Estévez-González et al. (2010) [[43](#_ENREF_43)] |
| TaqIB (rs708272)  (G > A) | A/G | Iranian  Patients with MetS  n = 80 | 38.91 ± 6.90 | Artichoke leaf extract (ALE) | Double-blind RCT: 1800 mg /day of ALE vs placebo for 12 weeks. | TC, HDL, LDL and TG | None of the SNP-diet interactions were statistically significant. | Rezazadeh et al. (2018) [[102](#_ENREF_102)] |
| TaqIB (rs708272)  (G > A) | A/ | Spanish  Participants at high risk of CVD  n = 650 | Unavailable | Mediterranean diet | Three diets: Mediterranean diet with olive oil; Mediterranean diet with nuts; and control (low-fat diet); 3 months. | Plasma lipids | None of the SNP-diet interactions were statistically significant. | Frances et al. 2006 (Abstract) [[42](#_ENREF_42)] |
| TaqIB (rs708272)  (G > A) | A/ | Brazillian  Participants with moderate primary Hypercholesterolemia  n = 60 | 20–60 | Plant sterol ester (PSE) | Double-blind cross-over: 20g /day margarine with (PSE) vs 20g /day margarine without PSE; 4 weeks. Food record. | TC, HDL, LDL and TG. | None of the SNP-diet interactions were statistically significant. | Lottenberg et al. (2003) [[103](#_ENREF_103)] |
| TaqIB (rs708272)  (G > A) | A/G | New Zealander  Men with  Hypercholesterolaemia  n = 85 | 48.5 ± 9.5 | Kiwifruit | RCT: 4-week healthy diet, followed by 4-week healthy diet vs healthy diet plus 2 kiwifruit per day. | TC, HDL, LDL and TG | In participants with 2 copies of the major allele (G), consumption of kiwifruit resulted in lower TG:HDL ratio than the control diet (mean change in TG:HDL ratio (mmol/l), -0.23 ± 0.58 vs 0.09 ± 0.56, P = 0.03; P_interaction_ < 0.05).  None of the other SNP-diet interactions were statistically significant. | Gammon et al. (2014) [[62](#_ENREF_62)] |
| TaqIB (rs708272)  (G > A) | A/A | Han Chinese  Healthy participants  n = 56 | 22.9 ± 1.8 | Carbohydrate and fat intake. | Washout diet of 31% fat and 54% carbohydrate for 7 days; followed by high carbohydrate/low fat diet (HC/LF) of 70% carbohydrate and 15% fat for 6 days. | TC, HDL, LDL and TG | After the HC/LF diet, carriers of the minor allele (A) had higher HDL (mean HDL (mg/dl): 56.14 ± 10.69 after washout diet vs 59.77 ± 10.62 after high carbohydrate/ low fat; P_interaction_ < 0.05).  None of the other SNP-diet interactions were statistically significant. | Du et al. (2010) [[58](#_ENREF_58)] |
| TaqIB (rs708272)  (G > A) | A/ | New Zealander  n = 70 | 48.25 ± 9.5 | Energy, carbohydrate, protein, total fat, SFA, PUFA, MUFA and dietary cholesterol. | Single crossover trial; high SFA vs high PUFA; two 4-week phases. | TC, HDL, LDL and TG. | None of the SNP-diet interactions were statistically significant. | Aitken et al. (2006) [[104](#_ENREF_104)] |
| TaqIB (rs708272)  (G > A) | A/ | Multi-ethnic (British, German, Danish and Italian)  n = 117 | 57.7 ± 5.4 | Isoflavone-enriched cereal bars. | Double-blind RCT: Cereal bars enriched with Isoflavone (genistein – to – daidzein ratio of 2:1; 50mg/day) or placebo cereal bars for 8 weeks with a washout period of 8 weeks before crossover. | TC, HDL, LDL and TG. | None of the SNP-diet interactions were statistically significant. | Hall et al. (2006) [[105](#_ENREF_105)] |
| TaqIB (rs708272)  (G > A) | A/ | Dutch  Healthy participants  n = 112 | 33 ± 16 | Rapeseed oil and plant stanol ester. | A low erucic acid rapeseed oil-based margarine and shortening for 4 weeks; followed by the same margarine (control) or 1 of 2 treatment groups: the same margarine and shortening + vegetable-oil-based plant stanol ester mixture or the same margarine and shortening + wood – based plant stanol ester mixture for 8 weeks. | HDL, LDL and TG. | None of the SNP-diet interactions were statistically significant. | Plat and Mensink (2002) [[106](#_ENREF_106)] |
| TaqIB (rs708272)  (G > A) | A/A | Greek  Men and postmenopausal women heterozygous for Familial  Hypercholesterolemia (HFH)  men: n = 41  women: n = 39  Healthy controls:  n = 11 | 44.37 ± 12.15 | Fatty meal | Oral fat tolerance test: 12-hour overnight fast followed by consumption of fatty meal within 20 minutes. | TC, HDL and TG | Among all participants, none of the SNP-diet interactions were statistically significant.  In HFH participants, women with the minor allele (A) had lower TG after 4 hours of fat intake (239 ± 65 vs 279 ± 95 mg/dl; P = 0.03) compared with men with the A allele. | Anagnostopoulou et al. (2009) [[60](#_ENREF_60)] |
| TaqIB (rs708272)  (G > A) | A/ | New Zealander  n = 55 | 45.5 ± 9.5 | SFA and PUFA. | Double cross-over: Lipid-lowering diet (baseline diet) for 3 weeks; high SFA vs high PUFA; 4 weeks. | TC, HDL, LDL and TG. | None of the SNP-diet interactions were statistically significant. | Wallace et al. (2000a) [[107](#_ENREF_107)] |
| TaqIB (rs708272)  (G > A) | A/ | New Zealander  n = 46 | 45.5 ± 9.9 | SFA and PUFA. | Double cross-over: Lipid-lowering diet (baseline diet) for 3 weeks; high SFA; high PUFA for 4 weeks. | TC, HDL, LDL, TG, light LDL and dense LDL. | None of the SNP-diet interactions were statistically significant. | Wallace et al. (2000b) [[108](#_ENREF_108)] |
| TaqIB (rs708272)  (G > A) | A/A | Dutch  Healthy participants  n = 405 | 29 ± 12 | SFA, trans fat, dietary cholesterol and coffee diterpenes cafestol and kahweol. | 7 trials with SFA; 2 trials with trans fat; 8 trials with dietary cholesterol; and 9 trials with coffee diterpenes cafestol and kaweol. | TC, HDL and LDL. | Participants with 2 copies of the minor allele (A) had higher changes in HDL in response to SFA (mean change in HDL (mmol): 0.08 ± 0.02 for ‘AA’, 0.03 ± 0.01 for ‘GA’, 0.04 ± 0.02 for ‘GG’ genotype; P = 0.04) than participants with ‘GG’ or ‘GA’ genotype.  Changes in LDL in response to dietary cholesterol were smaller in participants carrying the major allele (G) than those with AA genotype (mean change in LDL mmol/l: 0.27 ± 0.14 for ‘GG’, 0.35 ± 0.08 for ‘GA’, 0.75 ± 0.15 for ‘AA’; GG vs AA, P = 0.03; GA vs AA, P = 0.01).  None of the other SNP-diet interactions were statistically significant. | Weggemans et al. (2001) [[59](#_ENREF_59)] |
| rs5882 (I405V)  (G > A) | G/ A | Canadian  Sedentary men (12 pairs of monozygotic twins).  n = 24 | 21 ± 2.0 | Overfeeding. | Overfeeding by 1000Kcal /day, 6 days per week for a period of 100 days. | TC, HDL, LDL, VLDL, TG, HDL_2_ and  HDL_3_, | Overfeeding was associated with decreased HDL (mean change in HDL (mmol/l): -0.12 ± 0.04 vs 0.02 ± 0.04; P = 0.02), HDL_2_ (mean change in HDL_2_ (mmol/l), -0.08 ± 0.03 vs 0.03 ± 0.03; P = 0.04) and HDL_3_ (mean change in HDL_3_ (mmol/l), -0.04 ± 0.02 vs -0.004 ± 0.02; P = 0.002) in carriers of 2 copies of the major allele (A) compared to homozygotes for the minor allele (G).  None of the other SNP-diet interactions were statistically significant. | Terán-García et al. (2008) [[74](#_ENREF_74)] |
| rs5882 (I405V)  (G > A) | G/ | Brazillian  Participants with moderate primary hypercholesterolemia  n = 60 | 20–60 | Plant sterol ester | Double-blind cross-over: 20 g /day margarine with plant sterol ester (PSE) vs 20 g /day margarine without PSE for 4 weeks. Food record. | TC, HDL, LDL and TG | None of the SNP-diet interactions were statistically significant. | Lottenberg et al. (2003) [[103](#_ENREF_103)] |
| rs5882 (I405V)  (G > A) | G/ | Canadian  Individuals with mild hypercholesterolemia  n = 71 | 30 – 75 | Plant sterol | Dual centre single blind randomised crossover trial.  Margarine with 2g plant sterol/day vs margarine without plant sterol for 28-day periods. | TC, HDL, LDL and TG | None of the SNP-diet interactions were statistically significant. | MacKay et al. (2015) [[109](#_ENREF_109)] |
| rs5882 (I405V)  (G > A) | G/ | Iranian  Healthy participants  n = 85 | 20 ± 2 | PUFA and SFA | A high PUFA:SFA (1.2) followed by a low PUFA:SFA (0.3). Two consecutive 28-day periods. | HDL, LDL and TG | None of the SNP-diet interactions were statistically significant. | Darabi et al. (2009) [[110](#_ENREF_110)] |
| rs5882 (I405V)  (G > A) | G/ | Israeli  Healthy participants  n = 214 | 45.2 (14 - 74) | Energy intake, total fat, SFA and cholesterol. | Cross over: high SFA and cholesterol vs low SFA and low cholesterol. Two 4-week periods and a 4-week washout period. | TC, HDL, LDL and TG. | None of the SNP-diet interactions were statistically significant. | Friedlander et al. (2000) [[111](#_ENREF_111)] |
| rs5882 (I405V)  (G > A) | G/ | Greek  Men and postmenopausal women heterozygous for Familial  Hypercholestrolemia  men: n = 41  women: n = 39  Healthy participants:  n = 11 | 44.4 ± 12.2 | Fatty meal | Oral fat tolerance test: 12 hour overnight fast followed by consumption of fatty meal within 20 minutes. | TC, HDL and TG | None of the SNP-diet interactions were statistically significant. | Anagnostopoulou et al. (2009) [[60](#_ENREF_60)] |
| rs3764261  (C > A) | A/A | Spanish  ACS/CHD patients.  with MetS: n = 424 | 60.0 ± 0.3 | Mediterranean diet and low-fat diet. | 1-year dietary intervention involving Mediterranean diet (35% fat, 22% MUFA) vs Low-fat diet (28% fat, 12% MUFA). | TC, HDL, LDL and TG. | Intake of Mediterranean diet was associated with higher HDL (mean HDL (mg/dl): 41 vs. 38; P_interaction_ = 0.006) and lower TG (mean TG (mg/dl): 130 vs 146; P_interaction_ = 0.04) in participants carrying the minor allele (A) compared to those with CC genotype.  None of the other SNP-diet interactions were statistically significant. | Garcia-Rios et al. (2018) [[14](#_ENREF_14)] |
| rs3764261  (C > A) | A/C | US residents  White: n = 747  Black: n = 111  Hispanic, Asian or other: n = 36 | 51.0 ± 7.7 | High-fat diet, low-fat diet and carbohydrate. | 2-year randomised weight-loss trial (POUNDS LOST): Low-fat diet (20%) vs high-fat diet (40%)  n = 732  2-year RCT (DIRECT): Low-fat diet vs low carbohydrate (high fat) diet  n = 171 | TC, HDL, LDL and TG | Among participants with 2 copies of the major allele (C), those in the high-fat diet (40% fat) group had a higher increase in HDL (11.7 vs 4.5%; P_interaction_ = 0.01) and a larger decrease in TG (-25.1 vs. -11.7%; P_interaction_ = 0.0007) compared with those in the low-fat diet (20% fat) group.  None of the other SNP-diet interactions were statistically significant. | Qi et al. (2015) [[46](#_ENREF_46)] |
| C-629A (rs1800775)  (C > A) | C/ | Multi-ethnic  White: n = 395  South and South East Asian: n = 46  Black African: n = 38 | 51.5 ± 9.5 | SFA, MUFA, low fat, carbohydrate (CHO). | RCT: 4-week reference diet (∼18% SFA, 12% MUFA, 38% total fat, 45% CHO) followed by 1 of 3 diets: a MUFA diet (∼10% SFA, 20% MUFA, 38% total fat, 45% CHO); a low fat diet (∼10% SFA, 11% MUFA, 28% total fat, 55% CHO); or the reference diet for 24 weeks. | TC, HDL, LDL and TG | None of the SNP-diet interactions were statistically significant. | Walker et al. (2011) [[83](#_ENREF_83)] |
| rs9989419  (A > G / A > T) | A/ | Multi-ethnic  White: n = 395  South and South East Asian: n = 46  Black African: n = 38 | 51.5 ± 9.5 | SFA, MUFA, low fat, carbohydrate (CHO). | RCT: 4-week reference diet (∼18% SFA, 12% MUFA, 38% total fat, 45% CHO) followed by 1 of 3 diets: a MUFA diet (∼10% SFA, 20% MUFA, 38% total fat, 45% CHO); a low fat diet (∼10% SFA, 11% MUFA, 28% total fat, 55% CHO); or the reference diet for 24 weeks. | TC, HDL, LDL and TG | None of the SNP-diet interactions were statistically significant. | Walker et al. (2011) [[83](#_ENREF_83)] |
| C>T/In9 (rs289714)  (G > A / G > C) | C/ | Canadian  Sedentary men (12 pairs of monozygotic twins).  n = 24 | 21 ± 2.0 | Overfeeding. | Overfeeding by 1000Kcal per day, 6 days per week for a period of 100 days. | TC, HDL, LDL, VLDL, TG, HDL_2_ and  HDL_3_, | None of the SNP-diet interactions were statistically significant. | Terán-García et al. (2008) [[74](#_ENREF_74)] |
| rs173539  (C > T) | T/ | Multi-ethnic  White: n = 395  South and South East Asian: n = 46  Black African: n = 38 | 51.5 ± 9.5 | SFA, MUFA, low fat, carbohydrate (CHO). | RCT: 4-week reference diet (∼18% SFA, 12% MUFA, 38% total fat, 45% CHO) followed by 1 of 3 diets: a MUFA diet (∼10% SFA, 20% MUFA, 38% total fat, 45% CHO); a low fat diet (∼10% SFA, 11% MUFA, 28% total fat, 55% CHO); or the reference diet for 24 weeks. | TC, HDL, LDL and TG | None of the SNP-diet interactions were statistically significant. | Walker et al. (2011) [[83](#_ENREF_83)] |
| ^a^ Alleles are reported in the forward direction in line with dbSNP.  ^b^ P_interaction_ values are reported only for the significant SNP-diet interactions.  SNP ̶ single-nucleotide polymorphism; TC ̶ total cholesterol; HDL ̶ high-density lipoprotein cholesterol; LDL ̶ low-density lipoprotein cholesterol; VLDL ̶ very low-density lipoprotein cholesterol; TG ̶ triglycerides; SFA ̶ saturated fatty acids; PUFA ̶ polyunsaturated fatty acids; MUFA – monounsaturated fatty acids; n-6 PUFA – omega 6 polyunsaturated fatty acids; n-3 PUFA ̶ omega 3 polyunsaturated fatty acids; T2D ̶ type 2 diabetes; CHD – coronary heart disease; CVD – cardiovascular disease; MI – myocardial infarction; ACS ̶ acute coronary syndrome; RCT ̶ randomised controlled trial. | | | | | | | | |
